# Transcriptome driven characterization of curly- and smooth-leafed endives reveals molecular differences in the sesquiterpenoid pathway

**DOI:** 10.1038/s41438-018-0066-6

**Published:** 2019-01-01

**Authors:** Giulio Testone, Giovanni Mele, Elisabetta di Giacomo, Gian Carlo Tenore, Maria Gonnella, Chiara Nicolodi, Giovanna Frugis, Maria Adelaide Iannelli, Giuseppe Arnesi, Alessandro Schiappa, Tiziano Biancari, Donato Giannino

**Affiliations:** 1 Institute of Agricultural Biology and Biotechnology, Unit of Rome, National Research Council of Italy (CNR), Rome, Italy; 2 Department of Pharmacy, University of Naples Federico II, Napoli, NA Italy; 3 Institute of Sciences of Food Production, CNR, Bari, Italy; 4 Enza Zaden Italia, Tarquinia, Viterbo, Italy

**Keywords:** Transcriptomics, Secondary metabolism, Plant molecular biology

## Abstract

Endives (*Cichorium endivia* L.) are popular vegetables, diversified into curly/frisée- and smooth/broad-leafed (escaroles) cultivar types (cultigroups), and consumed as fresh and bagged salads. They are rich in sesquiterpene lactones (STL) that exert proven function on bitter taste and human health. The assembly of a reference transcriptome of 77,022 unigenes and RNA-sequencing experiments were carried out to characterize the differences between endives and escaroles at the gene structural and expression levels. A set of 3177 SNPs distinguished smooth from curly cultivars, and an SNP-supported phylogenetic tree separated the cultigroups into two distinct clades, consistently with the botanical varieties of origin (*crispum* and *latifolium*, respectively). A pool of 699 genes maintained differential expression pattern (core-DEGs) in pairwise comparisons between curly vs smooth cultivars grown in the same environment. Accurate annotation allowed the identification of 26 genes in the sesquiterpenoid biosynthesis pathway, which included several *germacrene* *A synthase*, *germacrene* *A oxidase* and *costunolide* *synthase* members (*GAS*/*GAO*/*COS* module), required for the synthesis of costunolide, a key precursor of lactucopicrin- and lactucin-like sesquiterpene lactones. The core-DEGs contained a *GAS* gene (contig83192) that was positively correlated with STL levels and recurrently more expressed in curly than smooth endives, suggesting a cultigroup-specific behavior. The significant positive correlation of *GAS*/*GAO*/*COS* transcription and STL abundance (2.4-fold higher in frisée endives) suggested that sesquiterpenoid pathway control occurs at the transcriptional level. Based on correlation analyses, five transcription factors (MYB, MYB-related and WRKY) were inferred to act on contig83192/*GAS* and specific STL, suggesting the occurrence of two distinct routes in STL biosynthesis.

## Diving into the genetics of endives

Genetic analyses show molecular differences that could explain why curly endives taste bitterer than smooth ones. Donato Giannino of the Institute of Agricultural Biology and Biotechnology and colleagues in Italy analyzed the genetic differences between curly and smooth endives, a leafy vegetable used in salads. They found more than 3000 single sequence variations in genes distinguishing the two types from each other. Twenty six genes were involved in the biosynthesis of sesquiterpenoids, metabolites important for plant survival that also contribute to the bitter taste of endives and have antimalarial, sedative and analgesic effects when isolated for humans. Their levels were higher in curly than in smooth endives, potentially contributing to their more bitter taste. The information expands the genetic data available on endives for breeding programs.

## Introduction

The *Cichorium endivia* (L.) species belong to the *Asteraceae* family and includes the botanical varieties *crispum* and *latifolium* (Lam.), which are respective sources (GRIN db, https://npgsweb.ars-grin.gov/gringlobal/search.aspx) of two market cultivar types (cultigroups), the curly- and smooth-leafed endives. The former (synonyms: frisée, cut-type) bear green leaves with a narrow central vein, septate blade with incised margins (syn.: runcinated-bipinnatifid type), while the smooth types (syn.: escaroles) produce lighter green leaves with a large midrib, a broad and slightly lobed lamina and dentate margins. Agronomic and some physiological features of the two cultigroups have been well-characterized^1^. The consumption of endives has been increasing in fresh and minimally processed segments worldwide and greatly in Europe, where Italy, Spain, and France are major representatives of *Cichorium* spp. products (TrendEconomy, http://trendeconomy.com).

*C. endivia* life cycle is annual and flowering extends from May to August at the Mediterranean latitudes; the leaf rosette (head) develops an inflorescence stem bearing violet autogamous flowers. Self-compatibility prevails, the outcross rate is 1%^2^ and leads to high inbreeding grade in natural populations that consist of a mixture of highly homozygous lines. Genetically, *C. endivia* (2n = 2x = 18) has a complex chromosomal organization^3^ and its genome size 1 is alleged to share  that of the close relative *C. intybus* in a range of 0.7–1.3 Gb^5,6^, it will be better defined after the genome sequence release. To date, the *Cichorium* spp. genetic consensus map^7^ has included markers from a *C. intybus* × *C. endivia* cross^4^; molecular marker assisted breeding of endive is expected to increase considering the recent development of genomic tools^8^. The strict autogamy compels the breeding strategies to mass or individual selections, pedigree breeding, and back-crossing^9^. Commercialized cultivars mostly consist of pure lines derived from repeated selfings of plants from local populations or of hybrids selected from parental line cross (F_1_ hybrid production is poorly explored ). Breeding programs are mainly performed by private seed companies to develop varieties suitable for the fresh-salad or minimally processed-salad markets, able to span the whole year cultivation (outdoor or in greenhouse), namely cold resistant in spring and heat tolerant in summer. Major traits targeted include resistance to premature bolting, tip burn, root rot and mildew, preservation/enhancement of nutritional quality, taste, and shelf life^1^.

Sesquiterpene lactones (STLs) are terpenoids with lactone rings, produced as secondary metabolites important for plant survival, typical of and used to classify *Asteraceae* species^10^, and known for exerting both positive and negative effects on human health^11^. Endive contains STLs, which can act on both nutraceutical and taste traits. The most abundant STLs of endive leaves are lactucin, 8-deoxylactucin, lactucopicrin, and the respective 11(S),13-dihydroderivatives^12^ though novel STLs have been discovered^13^. Lactucopicrin has been used as antimalarial, sedative, and analgesic in humans^14,15^ and recently as a protector against neurodegenerative diseases^16^. STLs also contribute to bitter taste, a crucial trait in terms of rejection or acceptance, which depends on consumers’ use and culture^17^. Bitterness has been associated with lactucin (Lc-) and lactucopicrin (Lp-) classes in chicory^18^ and lettuce^19^; in endives, Lp has a dominant effect on bitterness perception, though complex equilibria between STL and phenolics also exert a significant impact^20^. STLs belong to the germacrene A type sesquiterpenoids. The sequential actions of germacrene A synthase and oxidase, and costunolide synthase lead to costunolide, which is the precursor of STLs^21^. These enzymes and genes (*GAS, GAO*, *COS*) have been specifically characterized in *C. intybus*^21–24^ and not yet in *C. endivia*. Furthermore, the enzymes that use costunolide to synthesize both Lc- and Lp-like compounds have remained unknown in plants, so far.

The major aims of this work were to widen the knowledge on the differences between curly- and smooth-leafed endives by analyzing allelic and gene transcriptional variation as well as to investigate on gene divergences in the STL pathway contextually with the notion that curly types have higher STL contents than escaroles^20^. A reference transcriptome was assembled and annotated using the “Domari” curly cultivar. RNA-sequencing of five cultivars produced both SNP markers, which could neatly separate the two cultigroups into two distinct phylogenetic clades, and pools of up- and down-regulated genes (core-DEGs), which maintained the differential pattern in curly vs smooth genotypes. One core-DEG *GAS*, belonging to the 26 genes of the STL pathway, was recurrently more expressed in curly than smooth endives and positively correlated with STL abundances. Co-expression/correlations analyses based on biosynthesis genes/transcription factors expression and STL amounts supported that STL pathway control occurs at the transcriptional level; they also allowed inferring the roles of MYB, MYB-related and WRKY transcription factors on *GAS* regulation and the depiction of likely networks that subtend Lp- and Lc- compound synthesis.

## Material and methods

### Plant material, growth conditions, and sampling

The “Domari”, “Imari”, and “Myrna” are curly-leafed endives (*C. endivia* var. *crispum*); “Confiance” and “Flester” are smooth/broad-leafed types (*C. endivia* var. *latifolium*). The Enza Zaden company (www.enzazaden.com) owns these patented cultivars (www.cpvo.europa.eu) and provided seed lots.

Fields were located in Tarquinia, Lazio, Italy (42°15′N 11°44′E, 31 m a.s.l.); soil characteristics and cultivation parameters were previously reported^25^. In this work, plants were shown in nursery (3 dm^2^/well) at the end of August 2012; 3-week-old seedlings were moved into open field (8.2 plants/m^2^) and harvest occurred on the second half of November. The average temperature was of 18.4 ± 3.3 °C (www.idrografico.roma.it/annali). Details on agro-techniques (basal dressing, fertirrigation, protection vs weeds, thrips, moths and powdery mildew) are available upon request.

**Fig. 1:**
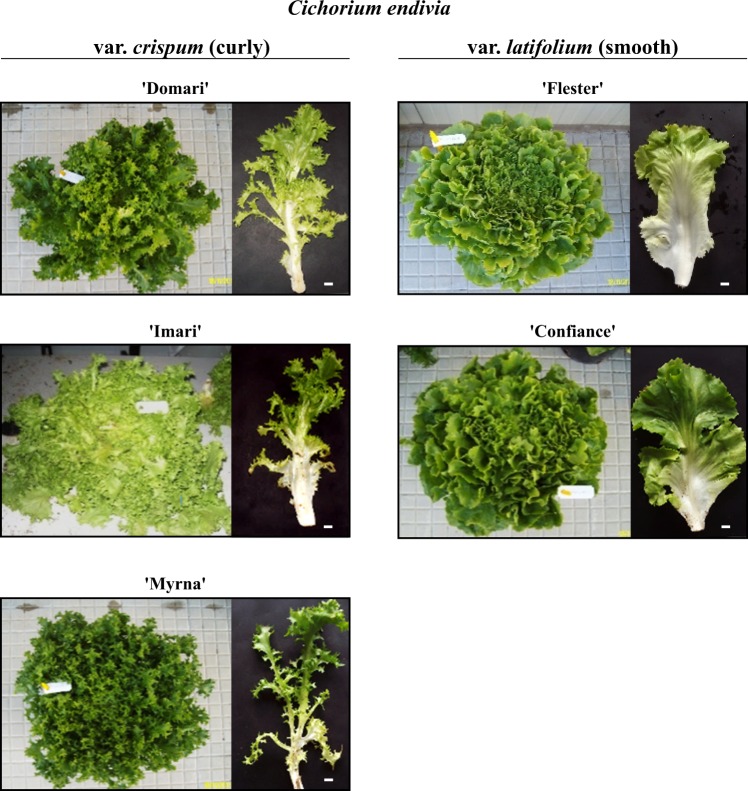
Phenotypes of endives at harvesting. Heads and respective leaf types used in the study of curled- and smooth-leafed cultivars (left and right columns). Bar size of leaf panel = 1 cm. Other morphometric parameters are listed in Table S1

Harvested heads (*n* = 9 per cultivar) were brought to laboratories and weighted (Fig. 1 and Table S1); the external leaves were removed from the rosette and the following leaves were sampled (because assumed as representative of freshly consumed or fresh-cut products). More precisely, 10 leaves were excised from each plant (*n* = 3) of the same cultivar and pooled to form a replicate batch (RB) of 30 leaves; these had comparable weights among the cultivars, though significant differences for length and surface were scored (Fig. 1 and Table S1). Three RB were rapidly generated (biological triplicates) and frozen in liquid nitrogen, gently crunched by hands and stored at −80 °C. The content of each RB was either used for RNA isolation in transcriptional and allelic variation analyses or further lyophilized at −50 °C for 72 h (lab freeze dryer with stoppering tray dryer, FreeZone®; Labconco Corp., Kansas City, MO, USA) and stored at −20 °C for STL quantification.

### RNA isolation, sequencing, and transcriptome assembly

For transcriptome reference assembly, ten “Domari” seedlings at the transplant (bearing 3–4 leaves) and ten plants at commercial maturation were selected. Apices, stems, leaves, or roots at the two developmental stages (*n* = 8) were used to isolate and purify total RNA (TRIzol, Invitrogen; RNAeasy kit, Qiagen). As for RNA sequencing, a mix of the eight samples (1 µg of total RNA each) was obtained; RNA yields and integrity (RIN > 7) were assessed (NanoDrop ND-1000, Thermo Scientific Inc; BioAnalyzer 2100; Agilent Technologies Inc.), cDNA libraries were synthesized (TruSeq RNA-seq kit, Illumina) and sequenced in 100 bp paired-end mode (Illumina HiSeq2000; IGA Technology Services, Udine, Italy). As for NGS transcriptional analyses and SNP mining, cDNA libraries were prepared from RNA of targeted leaves as described above and sequenced in 50 bp single-end. Three (“Myrna”, “Confiance”, “Flester”) and two (“Domari” and “Imari”) biological replicates were analyzed. RNA-seq datasets were stored in the National Centre for Biotechnology Information database (NCBI, www.ncbi.nlm.nih.gov) under the BioProject accession number PRJNA417356.

The transcriptome was assembled following the previously described one-step and two-step approaches^24^. Briefly, the output of one-step de novo assembly by Trinity v.2.2.0 ^26^ was merged with the two-step assembly obtained from an EST-based backbone plus a de novo assemblies by Velvet v.1.2.10/Oasis v. 0.2.08 ^27,28^. Subsequently, the redundancies were removed by TGICL-CAP3 v. 2.1 ^29^ and the transcript/isoform clustering was achieved by the CD-HIT package v. 4.6.6 ^30^ with an identity threshold of 97%, and the longest transcripts were counted as representative for each cluster. BLASTX (cut-off *E*-value ≤10^−5^) carried out annotation through these databases: Nr (NCBI non-redundant database; last update: 6 March 2017), RefSeq (NCBI Reference Sequence Database; release 79), TAIR10 (The Arabidopsis Information Resource, ver. 10), SwissProt and TrEMBL sections of the UniProt Knowledgebase (release-2017_05), KOG (euKaryotic Ortholog Groups)^31^. Full-length transcript analysis was carried out using the “analyze_blastPlus_topHit_coverage.pl” script from the Trinity package. Blast2GO 4.1 ^32^ was used to retrieve Gene Ontology (GO) and KEGG^33^ annotations from the best hits from BLASTX analysis. GO functional classification was achieved by WEGO^34^. KEGG pathway annotation was improved by mining KAAS (KEGG Automatic Annotation Server)^35^. Protein domain/families annotation was achieved by InterProScan 5.1-44.0 ^36^. Transcription factors (TFs) were predicted using the PlantTFDB v.4 ^37^. Multi-level quality evaluation of “Domari” transcriptome was achieved in three steps: (1) assessment of the number of reads that mapped back to the final assembly as proper-paired matches by the “bowtie_PE_separate_then_join.pl script” from Trinity package; (2) evaluation of assemblies against a plant database containing near-universal single-copy orthologue genes (BUSCO ver. 3)^38^; (3) estimation of the number of full-length transcripts against Nr database by the Perl script “analyze_blastPlus_topHit_coverage.pl” of Trinity.

### Polymorphisms calling, phylogenetic trees, and high-resolution melting (HRM) analysis

MIcroSAtellite identification tool v1.0 (MISA; http://pgrc.ipk-gatersleben.de/misa) was run to score simple sequence repeats (SSRs) and to target 1 to 6 nucleotide-long stretches using minimum repetitions (12 units for mono-, 6 for di-, and 5 for tri-, tetra-, penta-, and hexa-nucleotides). As for SNP mining, we used BWA v.0.7.15 ^39^, Picard tools v. 2.0.1 (http://broadinstitute.github.io/picard/), SAMtools v.0.1.19 ^40^, BamUtil v. 1.0.13 (https://github.com/statgen/bamUtil), and the bcftools utilities to, respectively, align reads to the transcriptome, mark duplicated reads, calculate genotype likelihoods, recalibrate base quality scores, and call variable positions. SNP reliability was enhanced by these filters: (a) quality score ≥30 (99.9% base call accuracy); (b) at least 10 high-quality reads supporting the nucleotide differences; (c) exclusion of SNPs within homopolymer stretches of length ≥5 bp; (d) genotype quality score ≥50. Cultivar-specific SNPs were concatenated into a FASTA sequence file to create phylogenetic tree by neighbor-joining method and MEGA6 software^41^.

GAS, GAO, and COS from *C. endivia* (Ce) and *C. intybus* (Ci) were submitted to GeneBank and the numbers from MG383453 to MG383471 were assigned. Protein phylogenetic analysis was carried out using the above-mentioned sequences together with the following ones: CiGASsh, AAM21659.1; CiGAO, ADF43080.1; CiCOS, AEG79727.1, and *Lactuca sativa* (Ls): LsGAS (LTC1), AAM11626.1; LsGAS (LTC2), AAM11627.1; LsGAS3, AOT80657.1; LsGAO1, D5J9U8.1; LsGAO2, AIX97103.1; LsCOS, AEI59780.1. The lettuce proteins marked with “Lsat” were retrieved from lettuce genome v.8 available at phytozome.jgi.doe.gov.

DNA was isolated by the DNeasy Plant Mini Kit (QIAGEN) and amplification and melt curve analysis were performed on Eco Real-Time PCR System (Illumina). The 10 µL reaction volumes included 10 ng of genomic DNA, 1× KAPA HRM FAST Master Mix (KAPA BIOSISTEMS), 0.2 µM of each primer (Tables S2) and 2.5 mM MgCl_2_. The reaction conditions were: enzyme activation at 95 °C for 3 min; 45 amplification cycles of 5 s denaturation at 95 °C and 30 s annealing/extension at 60 °C; final melting step at 95 °C, cooling to 60 °C and heating at 95 °C. Fluorescence data were collected every 0.1 °C from 60 to 95 °C. The melting curve were normalized between 100% and 0% fluorescent intensity by adjusting the pre- and post-melt normalization regions, respectively. Difference plots were generated by subtracting the normalized melting profiles against that of the “Domari” reference. The genotypes were discriminated visually from both normalized and difference melting curves.

### Digital gene expression (DGE) analyses and quantitative PCR (qPCR)

The single-end reads were mapped on the reference assembly by Bowtie2 (v. 2.2.9)^42^ and SAMtool pipeline, and read count for each transcript was scored in each replicate. The DGE levels were calculated and expressed as RPKM (Reads per kilobase per million mapped reads) values. Total RNA of leaf RBs was isolated (RNeasy Plant Mini Kit, Qiagen), DNase treated (RQ1, Promega), and 1 µg was reverse-transcribed at 55 °C by SuperscriptIII (Life Technologies). One microliter of a 1:10 cDNA dilution was amplified by Eco Real-Time PCR System (Illumina) using 1× Quantimix easy master mix (Biotools) and 0.3 µM of each primer (Table S2) in a 10 µl final volume. PCR reaction conditions: 95 °C for 10 min for polymerase activation, 45 cycles at 95 °C for 10 s, 60 °C for 30 s. The experiments included three biological and instrumental replicates. Gene expressions were normalized against the *ACT* reference gene^24^; mean normalized expressions and log2 fold change (log_2_ FC) were calculated by using the Q-Gene program^43^ and by the 2^−ΔΔCt^ method, respectively.

### STLs quantification

Total STL (comprising both free and bound fractions) were extracted by ultrasound assisted extraction^24^. Briefly, 2 g of lyophilized material was added to 50 mL of methanol/water solution (80:20, v/v) plus 2% of formic acid and 3 mL of santonin solution (101.7 µg/mL) as internal standard. The mixture was shaken and at 1000 g/min (F80 Digit, Falc Instruments s.r.l., Italy), for 15 min, at 80 °C. After collecting the supernatant, the pellet underwent two additional extractions as above. The final extract of 150 mL was vacuum-dried, re-dissolved in methanol/dichloromethane (1:7, v/v), and loaded onto a solid phase extraction (SPE) column. The elution was achieved with 6 mL of a dichloromethane/ethyl acetate (3:2 v/v) solution; subsequently, the eluted fractions were sonicated at 50 kHz for 30 min (37 °C) by an ultrasound bath (Labsonic LBS1-3, Falc Instruments s.r.l., Italy). The purified samples were added with methanol (4 mL) and the STL discrimination was achieved by an HPLC system (Thermo-Finnigan LLC, San Jose, CA), holding quaternary pump, DAD detector, and a C18 Kinetex column (250 × 4.60 mm, 5 µm). The mobile phases A and B were methanol/water 14:86 and 64:36 (v/v), respectively. The gradients were 0–20 min, 100–58% A; 20–30 min, 58% A; 30–45 min, 58–0% A; 45–50 min, 0% A; 50–52 min, 0–100% A; 52–62 min, 100% A. The flow was at 0.5 mL/min and the injection volume was 80 µL. STL peaks were determined at 260 nm (Fig. S1).

### Statistical analyses

ANOVA and Duncan Multiple Range Test were performed by Statistical Analysis System program (SAS software, Version 9.1, Cary, NC, USA). The principal component analysis (PCA) was based on mean centered and standardized data (unit variance scaled); results were pictured as bi-plots of scores (treatments) and loadings (variables) plots by using XLStat Pro (Addinsoft, Paris, France). As for DEG analysis, the Bioconductor edgeR package was used^44^. After sample normalization (based on trimmed mean of *M* values, TMM), unigenes with at least 1 read per million in at least three samples were selected; thresholds of gene expression difference significance were set on the co-occurrence of absolute value of log_2_ FC ≥1 and a false discovery rate (FDR) value ≤0.05. Finally, gene-metabolite correlation analyses were carried out by the R3.4.0 ^45^.

## Results

### Transcriptome features

A cDNA library was synthesized from equal quantities of RNA isolated from apical tips, stems, leaves, and roots of *C. endivia* plants sampled at both transplant and harvest stages (Table 1). The Illumina Hiseq2000 sequencing system generated approximately 246 million of raw reads (2 × 100 bp), which were processed to remove adaptors, ambiguous bases, and low-quality sequences, and 97.2% of them were retained for further processing (Table 1). Subsequently, the high-quality reads were assembled using two procedures as previously described^24^. The “one-step” procedure consisted of a de novo assembling by Trinity, which led to 255,105 sequences with an N50 and mean contig length of 1586 and 1048 bp, respectively (Table 2). The “two-step” pipeline included a template-based assembly followed by a de novo assembly. Briefly, the endive high-quality reads were first mapped on 30,170 EST of a public database (The *Compositae* Genome Project, http://compgenomics.ucdavis.edu) that produced 27,179 read supported sequences. These were subjected to iterative contig extension process (SeqMan Pro, DNAStar) that expanded the mean length from 753 to 1044 bp. The unmapped reads were retrieved by Bowtie2 and assembled de novo into 51,038 contigs by Velvet/Oasis. Finally, the outputs from one- and two-step pipelines were merged into a final “Domari” reference transcriptome of 84,882 transcripts (N50 = 1591 bp; average contig length = 1214 bp), including all isoforms, and clustered into 77,022 unigenes (Table 2).

**Table 1:** RNA-sequencing datasets

	**Reference**	**RNA-seq**				
Bot. variety	*crispum*	*crispum*			*latifolium*	
Cultivars	“Domari”	“Domari”	“Imari”	“Myrna”	“Confiance”	“Flester”
Tissues^a^	A, S, L, R	L	L	L	L	L
Stages^b^	T, H	H	H	H	H	H
Replicates	–	2	2	3	3	3
Read types	2 × 100 bp	1 × 50 bp	1 × 50 bp	1 × 50 bp	1 × 50 bp	1 × 50 bp
Raw reads	246,347,186	19,975,333	20,846,549	10,836,889	23,364,091	22,375,987
HQR (%)^c^	97.2	98.3	97.4	98.3	98.9	98.1

^a^*A*, apexes; *S*, stems; *L*, leaves; *R*, roots

^b^T, transplant; H, harvest

^c^High-quality reads, mean values for each group of replicates are reported

**Table 2:** Features of assembled transcriptomes

Transcriptome metrics	One-step assembly	Two-step assembly		Final assembly	
	*De novo* (Trinity)	EST-based	*De novo* (Velvet/Oases)	Transcripts^a^	Unigenes^b^
Sequence number	255,105	27,179	51,038	84,882	77,022
Sequence sizes (%)					
≤500 bp	35.9	17.8	19.9	19.0	17.5
501–1000 bp	24.7	37.4	35.5	32.1	31.7
1001–1500 bp	15.7	25.8	23.8	20.7	21.1
1501–2000 bp	10.6	12.2	11.9	13.2	13.8
2001–2500 bp	5.9	4.3	5.2	7.2	7.6
2501–3000 bp	3.2	1.7	2.1	3.6	3.8
>3000 bp	4.0	0.9	1.5	4.2	4.4
N50	1586.0	1258.0	1318.0	1591.0	1611.0
N90	469.0	577.0	566.0	605.0	623.0
Mean contig length (bp)	1048.0	1044.0	1065.2	1214.4	1235.2
Transcriptome size (Mb)	267.4	28.4	54.4	103.1	96.1
Read mapping back (%)					
Mapped	96.2	48.4	69.5	95.9	94.2
Properly paired	81.9	58.3	66.5	81.2	80.7
BUSCO evaluation (%)					
Completeness	89.9	20.2	58.7	89.8	89.6
Single copy	4.2	13.9	50.3	65.6	73.8
Duplicated	85.7	6.3	8.4	24.2	15.8
Fragmented	5.1	8.5	13.3	3.9	3.9
Missing	6.4	71.3	28.0	6.3	6.3

^a^Final output from the merge of one-step and two-step assemblies

^b^Contigs were clustered by CD-HIT; the longest transcripts were selected as representative for each isoform cluster (i.e. unigenes)

**Fig. 2:**
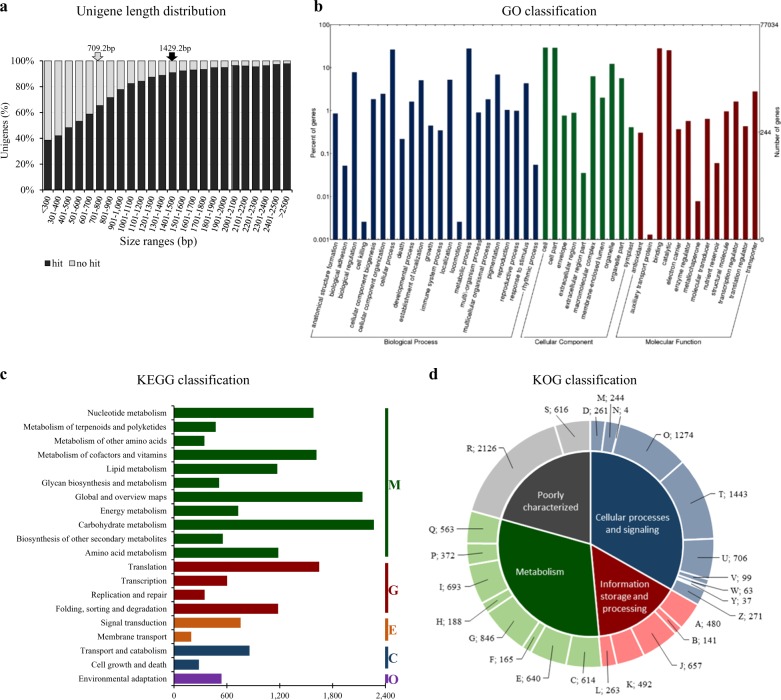
*C. endivia* unigene length distribution and annotation. **a** Unigenes distribution based on size (base pairs, bp) and BLASTX annotation. The black and gray arrows report the average length of annotated and non-annotated unigenes. **b** GO classification. The GO terms were classified into biological process (blue bars), cellular component (green bars), and molecular function (red bars). **c** KEGG classification; the histogram represents the unigene distribution into five major KEGG metabolic categories. M, metabolism; G, genetic information processing; E, environmental information processing; C, cellular processes; O, organismal systems. **d** Unigene functional classification into EuKaryotic Orthologous Groups (KOG). A, RNA processing and modification; B, chromatin structure and dynamics; C, energy production and conversion; D, cell cycle control, cell division, chromosome partitioning; E, amino acid transport and metabolism; F, nucleotide transport and metabolism; G, carbohydrate transport and metabolism; H, coenzyme transport and metabolism; I, lipid transport and metabolism; J, translation, ribosomal structure and biogenesis; K, transcription; L, replication, recombination and repair; M, cell wall/membrane/envelope biogenesis; N, cell motility; O, posttranslational modification, protein turnover, chaperones; P, inorganic ion transport and metabolism; Q, secondary metabolites biosynthesis, transport and catabolism; R, general function prediction only; S, function unknown; T, signal transduction mechanisms; U, intracellular trafficking, secretion, and vesicular transport; V, defense mechanisms; Y, nuclear structure; Z, cytoskeleton

As for annotation and function classification, the unigenes with at least one BLASTX hit were 57,579 (74.8 %) and showed average length of 1429.2 bp; non-annotated unigenes were 19,443 (25.2%) and of short size (Fig. 2a). BLASTX searches (*E*-value ≤10^−5^) against public protein databases showed that 73.0% unigenes had significant matches in the Nr database, the 74.0% in the TrEMBL, and the 70.8%, 67.1%, and 49.2% in the RefSeq, Tair, and SwissProt databases, respectively (Table S3). As for the functional categorization, the 52.7%, 20.0%, and 15.3% of unigenes were respectively annotated into GO, KEGG, and KOG (Table S3). Regarding GO clustering (Fig. 2b), the dominant subcategories were: “metabolic process” and “cellular process” in Biological Process (BP), “cell” and “cell part” in Cellular Component (CC), and “binding” and “catalytic activity” in Molecular Function (MF). Specifically, 27,935, 22,992, and 33,389, respectively, fell into BP, CC, and MF and 2371 had at least 10 GO terms (Fig. S2). As for KEGG categorization, 15,430 annotated genes were assigned to 131 pathways belonging to 20 metabolic groups (Fig. 2c); “carbohydrate metabolism” and “translation” represented the most abundant classes in “Metabolism” (M) and “Genetic information processing” (G), respectively. As for KOG (Fig. 2d), 11,783 annotated genes were assigned to 25 groups; the most abundant within the 4 macro-groups were: signal transduction mechanisms (T) in Cellular Process and Signalling; translation, ribosomal structure and biogenesis (J) in Information Storage and Processing; carbohydrate transport and metabolism (G) in Metabolism and General Function; and prediction only (R) in Poorly Characterized. In addition, 33,277 unigenes were annotated into InterPro database scoring 54,881 domains, 17,749 families, 3314 repeats, and 6444 functional sites (Table S3 and Fig. S3a); Protein kinase-like domain and Cytochrome P450 were the most represented in domains and families, respectively (Fig. S3b, c). Finally, 5475 sequences showed hits in plant transcription factor database (PlantTFdb, Table S3) and could be assigned to 57 families (Fig. S4a). The most represented were bHLH (9%), ERF (7%), C2H2 (6%), MYB-related, NAC and WRKY (5%), MYB, bZIP, and C3H (4%), and G2-like (3%).

Regarding the transcriptome quality (Table 2), the final assembly included ca. 81% of properly paired reads (out of ca. 94% of the reads that mapped back to the assembly), and completeness was ca. 90% according to BUSCO evaluation. In addition, 24,152 unigenes (43%, Table S4) were either full-length or nearly full-length transcripts, which had at least 70% of the alignment coverage to respective hits in the Nr protein dataset (Table S5). Overall, these data supported a satisfactory assembly, which included over 71% of single copy- and ca. 6% of duplicated genes (Table 2).

### Leaf-group differentiation based on sequence polymorphisms and gene expression

**Fig. 3:**
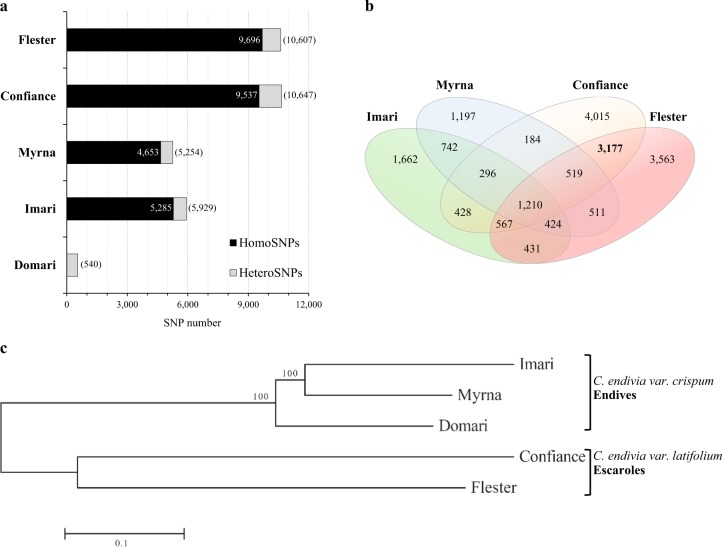
Single-nucleotide polymorphisms in curly and smooth cultivars. **a** The numbers of homozygous (black bars) and total SNPs are indicated; heterozygous SNPs are in gray bars. **b** The Venn diagram reports the number of SNPs specific to each cultivar or cultivar combinations (overlapping areas); the core-SNPs (see text) that typifies curly from smooth cultivars is in bold. **c** Genetic relationships among cultivars by concatenated SNPs (neighbor-joining method). Bootstraps values (at the branching points) are given for major nodes and are based on 1000 replicates. The length of the lines indicates the relative distances between nodes

Referring to the “Domari” transcriptome, 15,940 unigenes contained 19,951 putative SSRs and 3,155 unigenes had more than one microsatellite (Table S6). Neglecting the mononucleotides, the di- and tri-nucleotide repeats were the most abundant (respectively 51.4% and 45.2 % out of 9284 SSR) and the AG/CT and ATC/ATG were the most frequent motifs of these repeats (Table 3). After mapping the reads of cultivar against those of “Domari” transcriptome, total SNP numbers of “Imari”, “Myrna”, “Confiance” and “Flester” were 5929, 5254, 10,647, and 10,607, respectively (Fig. 3a). The homozygous SNPs were ca. 90% in each cultivar (compare black vs gray boxes) and “Domari” contained 540 hetero-SNPs. The SNP average frequency was of ca. 1/9000 bp for both “Confiance” and “Flester”, and 1/18,000 bp and 1/16,000 bp for “Myrna”, and “Imari”, respectively. The SNP number per unigene was greater in smooth than curly genotypes (“Confiance”, “Flester” vs “Imari”, “Myrna”); the former contained a mean of 0.14, which doubled that of the latter. Multiple pairwise comparisons allowed the identification of private SNPs (i.e. those that occur specifically in one population and not in all the others). Figure 3b reports a Venn diagram showing the number of cultivar-exclusive SNPs resulting from the different combinations. Overall, the number of private SNPs was highest in “Confiance” (4015), followed by “Flester” (3563), “Imari” (1622), and “Myrna” (1197); 3177 SNPs (core-SNPs) distinguished smooth vs curly cultivars. The core-SNPs spread over 1086 unigenes, 735 and 284 were respectively annotated into GO and KEGG (244 occurred in both), and these unigenes included 123 TFs. Enrichment analyses revealed the GO terms and KEGG pathways that were over-represented in the core-SNP gene set (Table S7). Moreover, concatenated SNPs were used to depict a genetic relationship tree (Fig. 3c), which placed endives and escaroles into two well separated clades. Finally, lab-scale SNP validation was achieved by an HRM technique performed on 16 randomly selected events. Of these, all used primer couples produced amplicons and the 97% confirmed the predicted polymorphism (Fig. S5 and Table S8).

**Table 3:** Summary of putative SSR in “Domari” unigenes

Unit repeat type	Number of repetitions							Total	Major type (%)
	5	6	7	8	9	10	>10		
Di-nucleotide	0	1289	864	649	637	521	813	4773	AG/CT (63.6%)
Tri-nucleotide	2301	1061	487	156	82	47	61	4195	ATC/ATG (24.4%)
Tetra-nucleotide	120	28	1	1	2	0	0	152	AAAT/ATTT (25.7%)
Penta-nucleotide	40	6	1	4	0	0	0	51	ACAGG/CCTGT (11.8%)
Hexa-nucleotide	59	17	11	4	3	5	14	113	AATGCT/AGCATT (6.2%)

**Fig. 4:**
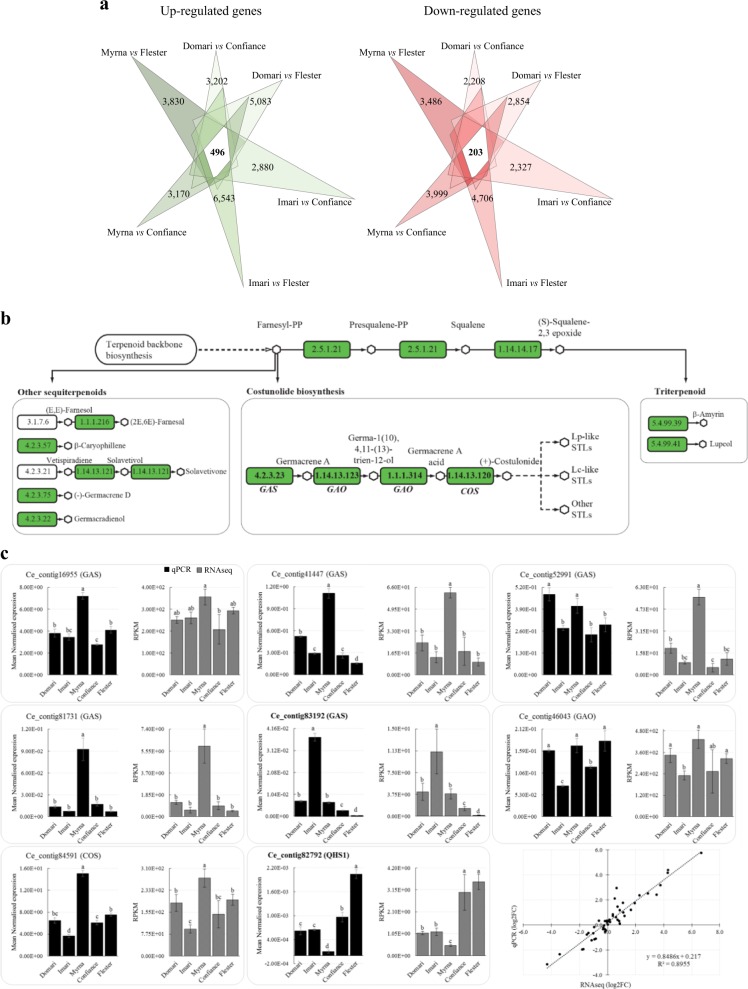
Differentially expressed gene analysis and STP biosynthesis pathway. **a** The Venn diagrams omit the numbers of differentially expressed genes in multiple comparisons (overlapping areas) and just report the number of up- (left) and down-regulated (right) genes specific to each comparison indicated at the vertexes. The number of genes that maintained the same differential transcription pattern in curly vs smooth cultivars (core-DEGs) is bolded. **b** Scheme of sesquiterpenoid and triterpenoid biosynthesis pathway in endive. Rectangles report the enzyme codes; and those assigned to endive contigs are in green (see Table 5). *GAS/GAO/COS* gene module acts in the costunolide synthesis branch. **c** Expression profiles of eight STP unigenes achieved by RNA-seq and qPCR (gray and black histograms, respectively). The unigene names in bold belong to the core-DEGs group. The last panel reports RNA-seq/qRT-PCR correlation analysis (bottom right) expressed as log2 fold change of curly vs smooth genotypes (six independent comparisons per gene); significant positive correlation occurred between the expression fold changes measured by the two methods (*R*^2^ = 0.89; *P* < 0.001)

Regarding gene expression, 496 and 203 genes were, respectively, up- and down-regulated in all the comparisons between endive vs escarole cultivars (Fig. 4a) and their merge (699 genes) is named core-DEGs. Moreover, KEGG enrichment analysis revealed nine pathways (Table 4) that contained over-represented core-DEGs. Among these, the sesquiterpenoid and triterpenoid (STP) biosynthesis pathway (Fig. 4b) included the Ce_contigs 83192 (*Germacrene A Synthase, GAS*) and 82792 (*Beta-Caryophyllene Synthase, QHS1*).

**Table 4:** KEGG pathway enrichment of core-DEGs

KEGG maps	DEGs^a^	Background^b^	FDR^c^	Rich factor^d^
map00943, Isoflavonoid biosynthesis	2 (2, 0)	13	9.16E-03	15.38%
map01040, Biosynthesis of unsaturated fatty acids	5 (4, 1)	43	7.28E-05	11.63%
map04712, Circadian rhythm - plant	10 (0, 10)	89	9.93E-09	11.24%
map00909, Sesquiterpenoid and triterpenoid biosynthesis	2 (1, 1)	26	2.38E-02	7.69%
map00670, One carbon pool by folate	2 (2, 0)	28	2.38E-02	7.14%
map00310, Lysine degradation	3 (1, 2)	79	2.38E-02	3.80%
map04110, Cell cycle	10 (6, 4)	283	1.51E-04	3.53%
map00970, Aminoacyl-tRNA biosynthesis	4 (4, 0)	138	2.38E-02	2.90%
map01110, Biosynthesis of secondary metabolites	6 (4, 2)	312	2.93E-02	1.92%

^a^Total number of DEGs in each KEGG map. The numbers of up- and down-regulated transcripts in curly vs smooth cultivars are in brackets. DEGs with KEGG annotation were 115

^b^Total number of unigenes in each KEGG map. Unigene with KEGG annotation were 15,431

^c^False discovery rate. The table includes pathways with values ≤0.05

^d^Ratio between the number of DEGs and unigenes annotated in a given pathway; higher values mean higher enrichment degree

### STP pathway: gene characterization and relationships with STL contents

Overall, the STP pathway included 26 unigenes encoding proteins ascribed to 11 distinct enzymes (Table 5, Fig. 4b). DGE analysis of edible leaves revealed that two unigenes were below the transcription threshold (RPKM 0–0.1), one was lowly expressed (RPKM 0.1–1), 12 showed moderate expression (RPKM 1–8), and 11 were highly expressed (RPKM > 8). The reliability of DGE analysis was confirmed by qPCR based on eight STP unigenes (Fig. 4c). The *GAS* and *QHS1* unigenes (Ce_contig83192 and 82792) showed respectively higher and lower expressions in curly than smooth genotypes. In order to enrich the gene pool of STP pathway, endive unigenes were blasted against the lettuce genome (phytozome.jgi.doe.gov) applying highly selective filters (identity ≥ 70%; full length ≥ 80%) and two two additional *GAO* (Ce_contig47698 and 11533) and two *COS* (Ce_contig69070 and 34331) orthologues were identified (Table S9).

**Table 5:** Unigenes in the sesquiterpenoid and triterpenoid pathway and DGE analysis

Biosynthetic pathways	EC codes	Description	Unigenes	Size (bp)	DGE (RPKM)^a^					ER^b^
					Domari	Imari	Myrna	Confiance	Flester	
Farnesyl and squalene	2.5.1.21	Squalene synthase (FDFT)	Ce_contig46083	1940	111.3 ± 4.0	78.6 ± 7.8	124.4 ± 0.5	120.9 ± 6.7	134.5 ± 7.1	H
	1.14.14.17	Squalene monooxygenase (SQLE)	Ce_contig50302	2164	61.9 ± 4.1	66.3 ± 7.5	38.0 ± 6.8	59.7 ± 1.7	49.8 ± 0.1	H
			Ce_contig83179	1808	0.8 ± 0.2	2.7 ± 1.3	0.3 ± 0.4	1.0 ± 0.0	0.7 ± 0.1	M
			Ce_contig85466	2036	5.0 ± 1.2	10.9 ± 2.5	6.9 ± 1.3	7.1 ± 2.5	6.4 ± 0.8	M
Costunolide	4.2.3.23	Germacrene A synthase (GAS)	Ce_contig16955	2898	251.3 ± 15.3	260.4 ± 26.7	355.6 ± 35.9	207.8 ± 67.4	293.1 ± 15.3	H
			Ce_contig41447	1874	24.2 ± 6	13.3 ± 4.2	61.9 ± 3.9	17.8 ± 10.4	9.7 ± 3.1	H
			Ce_contig52991	1903	19.3 ± 3.6	8.9 ± 1.1	56.0 ± 5.7	5.3 ± 3.1	11.4 ± 4.5	H
			Ce_contig81731	1079	1.2 ± 0.2	0.6 ± 0.3	5.3 ± 2.5	0.9 ± 0.3	0.5 ± 0.1	M
			**Ce_contig83192**	**1901**	**4.2** **±** **1.5**	**11.1** **±** **3.8**	**3.8** **±** **0.9**	**1.3** **±** **0.3**	**0.2** **±** **0.1**	M
	1.1.1.314 1.14.13.123	Germacrene A oxidase (GAO)	Ce_contig46043	2064	343.4 ± 40.4	231.1 ± 24.4	433.4 ± 50.9	253 ± 120.6	324.2 ± 26.8	H
	1.14.13.120	Costunolide synthase (COS)	Ce_contig84591	1984	187.5 ± 29.8	95.3 ± 14.5	275.6 ± 32.4	147.3 ± 47.7	198.6 ± 19.4	H
Other sesquiterpenoids	1.1.1.216	NADP+-farnesol dehydrogenase (FLDH)	Ce_contig25141	1385	30.7 ± 0.8	28.73 ± 3.8	24.7 ± 0.4	26.6 ± 1.0	26.9 ± 0.2	H
			Ce_contig25142	2085	3.7 ± 0.2	3.79 ± 3.6	3.6 ± 0.5	2.6 ± 0.7	3.1 ± 0.6	M
			Ce_contig57512	952	3.4 ± 0.9	1.96 ± 0.1	2.6 ± 0.3	2.4 ± 0.8	2.6 ± 0.3	M
			Ce_contig64598	597	15.7 ± 1.0	7.03 ± 1.9	15.6 ± 2.0	11.7 ± 1.7	13.0 ± 1.8	H
	4.2.3.75 4.2.3.22	Germacrene D synthase (GERD)	Ce_contig9237	974	0.0 ± 0.0	0.0 ± 0.0	0.0 ± 0.0	0.0 ± 0.0	0.0 ± 0.0	N
	1.14.13.121	Premnaspirodiene oxygenase (HPO)	Ce_contig34331	1267	3.8 ± 0.6	1.64 ± 0.1	9.6 ± 2.2	3.0 ± 0.3	3.6 ± 0.6	M
			Ce_contig54036	783	0.2 ± 0.1	0.16 ± 0.1	9.2 ± 0.3	4.3 ± 1.2	6.9 ± 0.7	M
			Ce_contig69070	1693	5.2 ± 0.1	2.33 ± 0.3	13.4 ± 1.5	3.4 ± 0.2	4.7 ± 0.3	M
	4.2.3.57	β-Caryophyllene synthase (QHS1)	**Ce_contig82792**	1984	**1.1** **±** **0.1**	**1.14** **±** **0.2**	**0.5** **±** **0.1**	**3.0** **±** **0.8**	**3.5** **±** **0.4**	**M**
Triterpenoids	5.4.99.39	β-Amyrin synthase (LUP4)	Ce_contig4595	523	0.0 ± 0.0	0.0 ± 0.0	0.0 ± 0.0	0.0 ± 0.0	0.0 ± 0.0	N
			Ce_contig16172	2965	100.3 ± 2.7	86.16 ± 13.1	133.6 ± 27.5	107.3 ± 10.7	93.4 ± 13.9	H
			Ce_contig72369	1602	1.8 ± 0.4	2.07 ± 0.9	1.8 ± 0.4	1.44 ± 0.5	1.3 ± 0.3	M
			Ce_contig73522	2434	13.9 ± 3.2	6.13 ± 0.2	13.8 ± 2.3	12.0 ± 0.4	9.7 ± 1.9	H
			Ce_contig86255	2633	0.1 ± 0.1	0.20 ± 0.2	0.1 ± 0.0	0.2 ± 0.0	0.1 ± 0.1	L
	5.4.99.41	Lupeol synthase (LUP1)	Ce_contig2965	2602	2.8 ± 0.3	2.99 ± 0.1	1.3 ± 0.2	1.9 ± 0.3	1.5 ± 0.4	M

^1^DGE, digital gene expression (mean ± standard deviation). Bolded values indicate differentially expressed unigenes (FDR = 0.05; |log2 fold change| = 1) in each curly vs smooth comparison

^b^ER, unigene mean expression range across all samples. H, high (RPKM > 8); M, moderate (RPKM 1–8); L, low (RPKM 0.1–1) expression. N, below the expression threshold (RPKM 0–0.1)

**Fig. 5:**
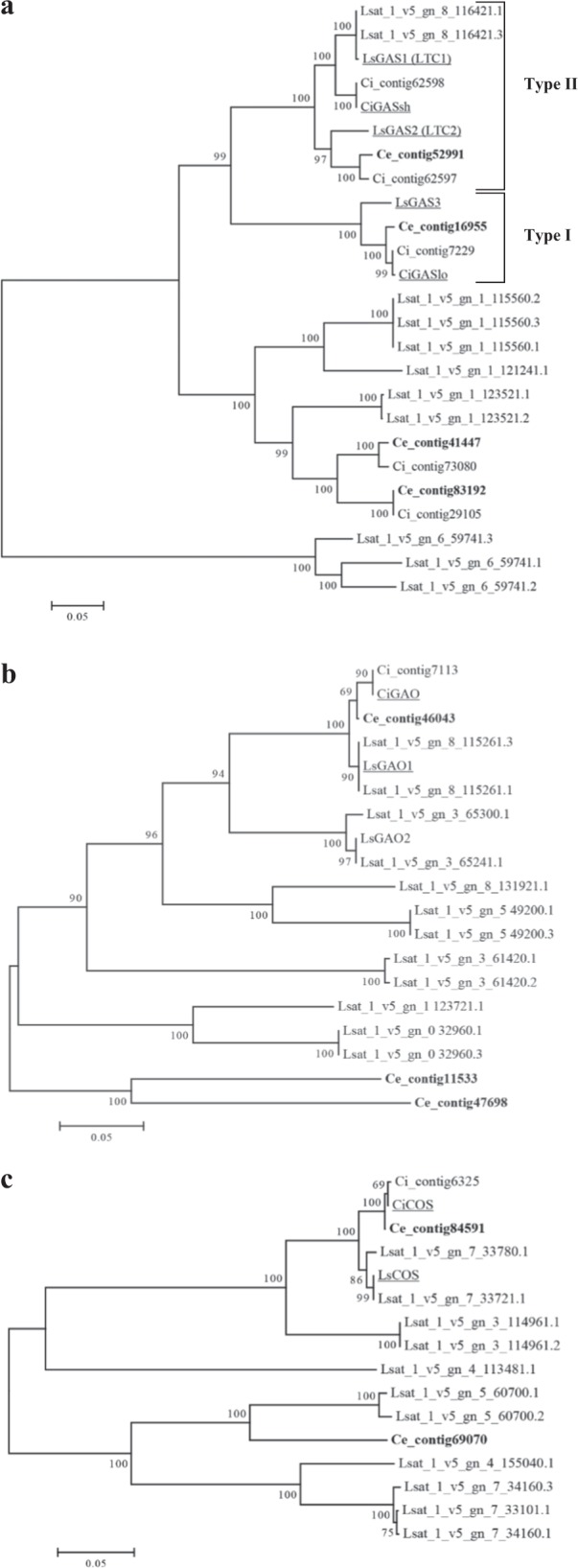
Phylogenetic analysis of STL biosynthesis proteins in the *Cichorieae* tribe. **a–c** Phylogenetic trees of germacrene A synthase (GAS), oxidase (GAO), and costunolide synthase (COS). They were constructed by neighbor-joining method, using deduced proteins of *Cichorium endivia* (Ce) contigs (in bold) and those retrieved from public databases of *Lactuca sativa* (Lsat or Ls) and *Cichorium intybus* (Ci) species. Bootstraps values were based on 1000 replicates. The line lengths indicate the relative distances between nodes. Underlined proteins have biochemically assessed functions (references in the text)

Phylogenetic trees (Fig. 5) of GAS, GAO, and COS proteins were constructed by using sequences from *Cichorium* spp. and *Lactuca sativa* of the *Cichorieae* tribe and excluding partial sequences (Ce_contig81731/GAS and Ce_contig34331/COS, Table S9). The analysis showed that new protein encoded by the Ce_contig16955 belonged to the type I GAS (Fig. 5a), which has lettuce LsGAS3 and chicory CiGASlo as reference proteins due to their assessed enzymatic function^23,46^. The Ce_contig52991 belonged to type II GAS, which have LsGAS1 and 2 and CiGASsh^23,47^ as references. The Ce_contig83192 and 41447, which shared 89.7% sequence identity (Fig. S6), were in a *Cichorium* spp. cluster sited near that of lettuce GAS enzymes with uncharacterized function. As for GAO (Fig. 5b), the Ce_contig46043 fell in the *Cichorium* spp. sub-group of CiGAO near that of lettuce LsGAO1, both with ascertained functions^48,49^. The Ce_contigs 11533 and 47698 formed a group per se. As for COS (Fig. 5c), the Ce_contig84591 was within a sub-group of *Cichorium* spp. having CiCOS and LsCOS as landmarks^48,50^, whereas the Ce_contig69070 formed a separate group. As for polymorphic events, cultivar-specific SNPs (Table S10) were scored in five non-differentially expressed genes (Ce_contigs: 16955/*GAS*, 52991/*GAS*, 46043/*GAO*, 69070/*COS*, and 84591/*COS*) from all cultivars, except for “Myrna”; finally, silent type substitutions prevailed.

The major STLs lactucin (Lc), 8-deoxylactucin (dLc), lactucopicrin (Lp) and the respective dihydroderivatives, 11(s),13-dihydrolactucin (DHLc), 11(s),13-dihydro-8-deoxylactucin (DHdLc), and 11(s),13-dihydrolactucopicrin (DHdLp) were quantified in edible leaves (Table 6). Globally, the total STL content (STLTOT) was significantly higher in curly- than smooth- endives (2239 ± 531 vs 930.8 ± 181.7 mg/kg dry matter) consistently with both total amounts of lactucin-like and lactucopicrin-like compounds (LcTOT, 1453.5 ± 548.5 vs 584.1 ± 73.8; LpTOT, 786.4 ± 186.8 vs 346.7 ± 113.1) and the mean abundance of each STL molecule. The STLTOT, LpTOT, and LcTOT also differed significantly among all the cultivars though overlapping values occurred in some specific STL compounds (e.g.: dLc contents of “Imari” vs “Flester”, Lp contents of “Domari” vs “Confiance”). The conversion of STL amounts into bitterness-deduced values indicated that curly endives had higher scores than escaroles (Table S11).

**Table 6:** Sesquiterpene lactone contents in leaves of curly and smooth endives

	STLs content (mg/kg dry matter)^a,b^								
Cultivars	Lc	DHLc	dLc	DHdLc	LcTOT	Lp	DHLp	LpTOT	STLTOT
“Domari”	580.2 ± 17.6b	295.3 ± 13.1a	154.3 ± 10.5c	351.9 ± 16.8b	1381.7 ± 16.3b	412.3 ± 18.3c	142.4 ± 10.1a	554.7 ± 27.7c	1936.4 ± 37.1b
“Imari”	474.5 ± 16.7c	206.5 ± 14.7b	98.5 ± 8.6d	80.4 ± 4.3e	859.9 ± 40.2c	976.9 ± 21.6a	1.0 ± 1.0e	977.9 ± 20.7a	1837.8 ± 21.8c
“Myrna”	695.2 ± 17.0a	180.5 ± 15.3b	835.3 ± 18.8a	407.8 ± 15.6a	2118.8 ± 37.9a	753.1 ± 20.4b	73.4 ± 4.5b	826.5 ± 20.4b	2945.3 ± 53.2a
“Confiance”	154.5 ± 11.3d	98.2 ± 10.6c	191.5 ± 14.2b	202.1 ± 12.1c	646.3 ± 7.7d	400.3 ± 15.4c	49.1 ± 6.3c	449.4 ± 9.6d	1095.7 ± 2.3d
“Flester”	160.5 ± 10.8d	113.1 ± 11.3c	101.0 ± 10.5d	147.3 ± 11.5d	521.9 ± 44.1e	214.6 ± 14.1d	29.3 ± 1.2d	243.9 ± 15.3e	765.8 ± 29.9e
*Significance* ^3^	***	***	***	***	***	***	***	***	***
Curly type	583.3 ± 96.7a	227.4 ± 53.6a	362.7 ± 355.5	280.0 ± 152.1	1453.5 ± 548.5a	714.1 ± 246.8a	72.3 ± 61.5	786.4 ± 186.8a	2239.8 ± 531.9a
Smooth type	157.5 ± 10.4b	105.7 ± 12.8b	146.3 ± 50.8	174.7 ± 31.8	584.1 ± 73.8b	307.5 ± 102.6b	39.2 ± 11.6	346.7 ± 113.1b	930.8 ± 181.7b
*Significance*	***	***	n.s.	n.s.	**	**	n.s.	***	***

^a^Lc lactucin, *DHLc* 11(S),13-dihydrolactucin, dLc 8-deoxylactucin, DHdLc 11(S),13-dihydro-8-deoxylactucin, Lp lactucopicrin, DHLp 11(s),13-dihydrolactucopicrin, LcTOT total lactucin-like STLs, LpTOT total lactucopicrin-like STLs, STLTOT total STLs

^b^Means marked with the same letters were not significantly different after the ANOVA and HSD Tukey’s test

^c^*, **, *** = significant at *P* < 0.05, 0.01, and 0.001, respectively. *n.s.* non-significant

**Fig. 6:**
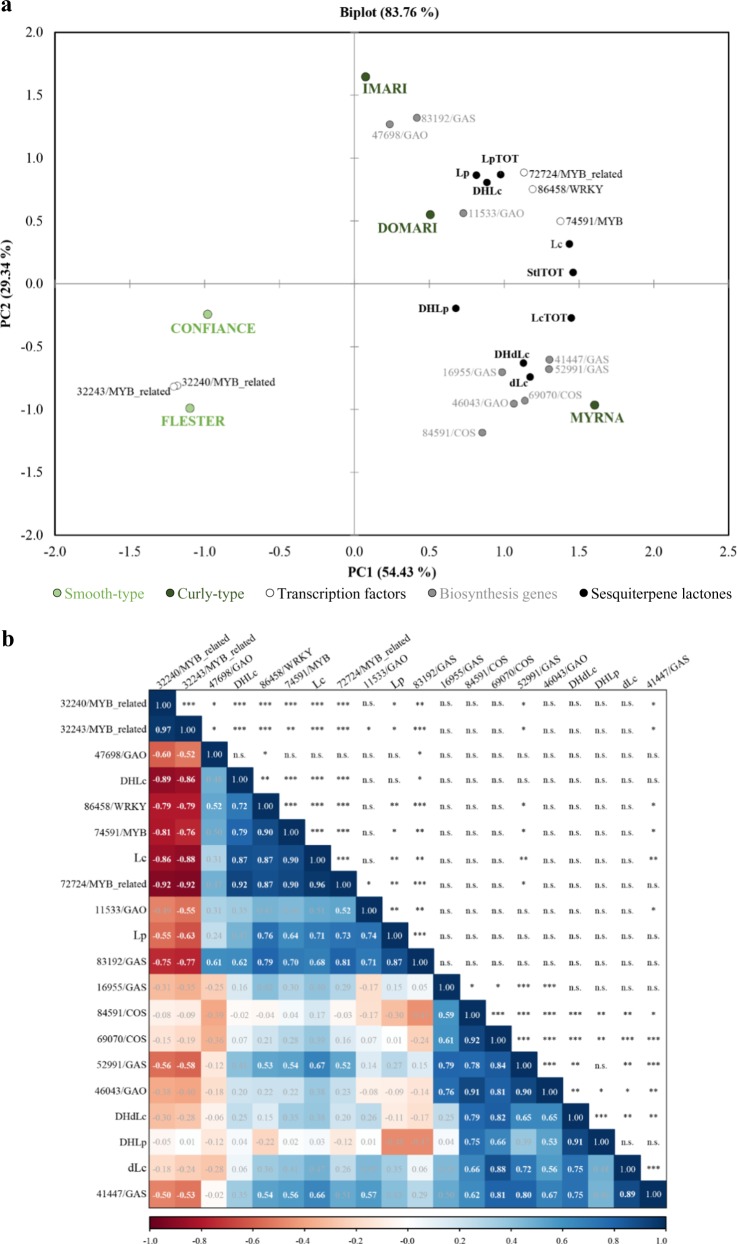
Principal component analysis and correlation plot in endive STL pathway. **a** PCA biplot of STL contents and biosynthesis/transcription factor gene expressions in curly- and smooth-leafed endives. *GAS* *germacrene A synthase*, *GAO* *germacrene A oxidase*, *COS* *costunolide synthase*. Lp lactucopicrin, Lc lactucin, DHLc 11(S),13-dihydrolactucin, dLc 8-deoxylactucin, DHdLc 11(S),13-dihydro-8-deoxylactucin, DHLp 11(s),13-dihydrolactucopicrin. **b** The Pearson’s coefficient (*r*) and correlation significance (asterisks) are disposed in a symmetric matrix made with the same variables as in PCA. The heat map places variables in hierarchical clustering; negative and positive correlations assign (*r*) values in red and blue squares, respectively; bold values refer to significant correlations. *, **, *** = significant at *P* ≤ 0.05, 0.01, and 0.001, respectively; n.s. non-significant

**Fig. 7:**
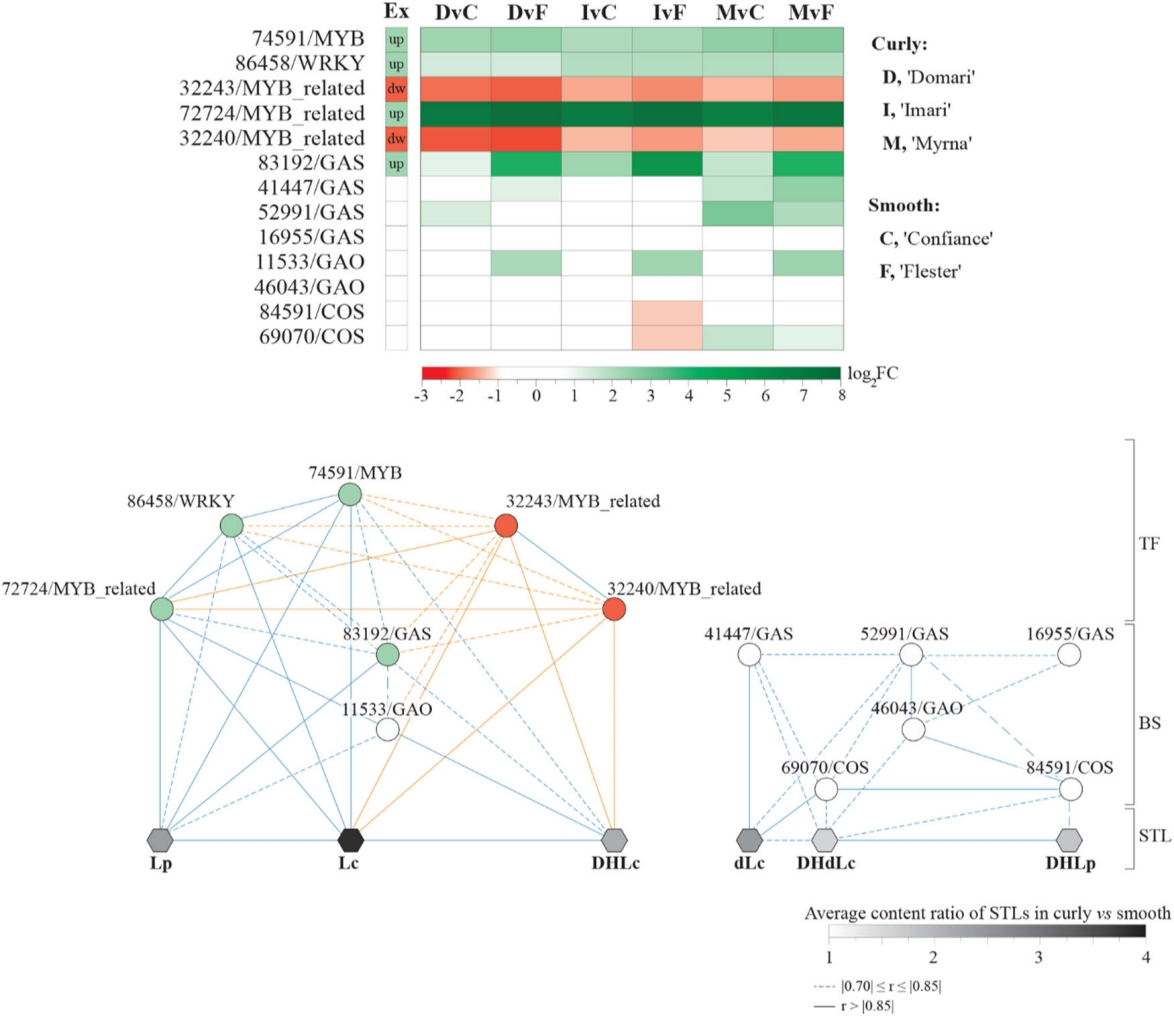
Putative network subtending synthesis of Lp- and Lc-like compounds. **a** Heat map visualization of the relative gene expression levels (log2 fold change) in curly vs smooth endives. The column “Ex” reports the up- or down-regulation expression pattern that was maintained in each curly vs smooth pairwise comparison (log_2_FC and FDR values are listed in Table S13). **b** Blue and orange edge represent positive and negative correlations, respectively. Solid or dashed traits refers to correlation strength according to the *r* coefficient ranges (bottom right). Transcription factors were sited upstream the *GAS-GAO-COS* biosynthesis gene module leading to sesquiterpene lactones. TF transcription factors, BS biosynthesis genes, STL sesquiterpene lactones; explanatory notes for STL abbreviations are in the legend of Fig. 6

After scoring differences in STL biosynthesis gene transcriptions and contents between the curly and smooth cultivars, we carried out a search for TFs that could be involved in pathway regulation. Several families of TF were identified in the core-DEGs, and the MYB-related and CO-like ones were the most numerous (Fig. S4b). Subsequently, overall exploration of data was approached by PCA focusing on correlations among *GAS/GAO*/*COS* biosynthesis (*BS*) and TF gene expressions and STL contents from all cultivars. The criteria to select TF genes from the core-DEGs included transcript completeness (≥80%), protein identity (≥70%), and inferred involvement in STP pathway based on putative functional analogies with well-characterized orthologues. These thresholds led to identify five TFs (Table S12). The biplot picture (Fig. 6a) showed that the PC1 explained 54.4% of the variation; the contents of all STLs, the expression of all *BS*, and three *TF* genes (Ce_contigs: 72724/*MYB-related*, 74591/*MYB*, and 86458/*WRKY*) of the curly cultivars (“Domari”, “Imari”, and “Myrna”) fell in the PC1 positive values. Oppositely, the smooth cultivars (“Flester”, “Confiance”) were on the PC1 negative side together with two *MYB-related* *TF* (Ce_contig32243 and 32240). The PC2 explained 29.3% of the variation highlighting those variables that separated “Myrna” (PC2 negative values) from “Imari” and “Domari” (PC2-positive values) within the curly group. “Imari” and “Domari” (top right quadrant) clustered with the LpTOT and a set of *GAS* (Ce_contig83192), *GAO* (Ce_contig47698 and 11533), and *TF* (Ce_contigs: 72724/*MYB-related*, 74591/*MYB* and 86458/*WRKY*) genes, diverging from the group (bottom right quadrant) made of “Myrna”, LcTOT, and six *BS* genes (Ce_contigs: 41447, 52991, and 16955/*GAS*; Ce_contig46043/*GAO*; Ce_contigs: 84591, 69070/*COS*). The grouping of gene expression and compound contents pinpointed at the occurrence of correlations further addressed by Pearson’s analysis (Fig. 6b) and hereafter we refer to those that have *r* ≥ |0.7| and *P* ≤ 0.01 as thresholds. The 83192/*GAS* and 86458/*WRKY* transcriptions were positively correlated, and each of the two had positive correlation with total lactucopicrin-like contents. The two *MYB-related TF* (Ce_contig32240 and 32243) had negative correlation with LpTOT (Fig. 6a, bottom left quadrant). Furthermore, the expressions of the six *BS* were positively correlated with DHdLc and dLc molecule contents (Fig. 6a, bottom right quadrants). No significant correlations occurred between *TF* and these *BS* genes. The analyses allowed the depiction of a putative gene/metabolite network into distinct branches (Fig. 7): one encompassed all *TF*, the *BS* genes 83192/*GAS* and 11533*/GAO*, and the Lp, Lc, and DHLc molecules; the other embraced the remaining *GAS, GAO,* and *COS* genes and the dLc, DHdLc, and DHdLp compounds.

## Discussion

In the current scenario where the number of *Asteraceae* spp. sequenced genomes has increased for high profit crops^51–53^, the endive transcriptome mining of this work has been a sustainable strategy aimed to gene finding, expression analysis, and marker production. The “Domari” transcriptome assembly was achieved through a pipeline that combined the template-based methods accuracy and the ability of de novo assemblers to detect novel transcripts. The strategy was confirmed to be convenient^24^ and led to a final transcriptome with better parameters than those produced by the separate use of the one- and two-step approaches, achieving higher N50 and mean contig length, reduced duplication and fragmentation events, and high levels of completeness and reads representation. The transcriptome of *C. endivia* var. *crispum* widens the number of those available in the species ^8^, though it differs for higher contig number, longer unigenes, and wider range of tissues from which RNA was isolated. Finally, it expands gene mining because it represents different vegetative tissues of young and ready-to-market plants and adds information on a recurrent parent cultivar.

The “Domari” transcriptome and cultivar re-sequencing provided a pool of SNPs that effectively fingerprinted the frisée and escarole cultigroups, respectively associated to the botanical var. *crispum* and *latifolium*^54^. Previously, the cultigroup classification was not fully supported by AFLP-marker analysis^55^, while the SNP-based phylogenetic analyses of this work neatly separated curly from smooth endives, supporting the cultigroup/taxa association, and  provided tools for cultivar traceability. Moreover, this new SNP pool may turn useful to better characterize genetic differences between *C. endive* and *C. intybus* species, which share introgression and complex relatedness^56,57^. Contextually, *C. endive* cultivars showed lower SNP average frequency than that measured in the *C. intybus* (1/9000–1/18,000 vs 1/1068 bp) transcriptome^24^, which may reflect the prevalent cross-fertilization in the latter species^58^. The homozygous SNPs were ca. 90% in all endive cultivars, likely due to breeding process based on repeated self-fertilizations. Finally, the production of SNPs specific for parental cultivars provides useful tools to create endive specific genetic maps made of expressed genes .

A set of 699 of unigenes (core-DEGs) maintained a leaf-group specific transcription pattern within over five thousand differentially expressed genes in all endive vs escarole comparisons. The core-DEGs fell in nine over-represented pathways (Table 4), including those of circadian rhythm and STP biosynthesis, and the *MYB-related* and *CO-like* transcripts appeared as the most numerous TFs within the core-DEGs (Fig. S4). The MYB-related and CO-like TFs are involved in circadian clock and photoperiod networks that control flowering time^59^. The common down-regulation of these genes in curly vs smooth endives may reflect leaf-group specific responses to growth cycle conditions and regulation of bolting time, a major breeding trait of *Cichorieae* leafy crops^58^. As for the STP biosynthesis pathway, the *QHS1* gene, putatively encoding an enzyme that catalyzes the synthesis of β-caryophyllene, maintained the differential expression pattern in curly- vs smooth cultivars and was up regulated in the former. β-caryophyllene is one of the most widespread sesquiterpene floral volatiles that acts in defense mechanisms^60^ and the *QHS1* expression pattern stimulates the speculation that different contents of β-caryophyllene, naturally found in *Cichorium* spp.^61^, may distinguish the leaf cultigroups and subtend different responses to biotic stress.

The costunolide biosynthesis branch of endive transcriptome consisted of 5 *GAS*, 3 *GAO*, and 3 *COS* transcripts that had significant sequence variability to suggest their origins from distinct genes. Consistently, in lettuce, each of these genes belong to families scattered in the genome and is able to encode isoforms by inferred alternative splicing (data retrievable from phytozome.jgi.doe.gov). The phylogenetic tree based on chicory, endive, and lettuce sequences highlighted the clustering of *C. intybus* and *C. endive* deduced proteins, confirming the species vicinity^54^. Some BS proteins (e.g. 52991 and 16955/GAS) fell in groups including members with ascertained function (Fig. 5) and they are likely to conserve it. However, the roles of other BS enzymes (e.g. GAS contigs: 83192 and 41447) need investigation, considering that amino acid stretch diversity (examples for GAS are in Fig. S6) suggests the occurrence of variation of catalytic functions and/or substrate specificity.

The PCA outcomes separated curly- from smooth types, the former were in association with STL contents and the expression of *BS*, *MYB,* and *WRKY* TFs, while the latter just grouped with two *MYB-related* factors (Fig. 6a). A set of *BS* genes (3 *GAS*, 1 *GAO* and  2 *COS*) showed positive correlation among themselves and vs the contents of DHdLc and dLc (Fig. 6b). Interestingly, DHLp was more significantly related to DHdLc/dLc than Lp/DHLc molecules (Fig. 6a) and showed significant positive correlation with just two *COS* and one *GAO* genes (Fig. 6b). Positive correlations between *BS* gene expression and STL contents were observed in chicory and artichoke^24,62^. Consistently, the higher expression of 41,447 and 52,991/*GAS* genes (Fig. 4c, qPCR panels) may explain the higher contents of dLc and DHdLc in the curly “Domari” and “Myrna” vs the smooth “Confiance” and “Flester”, while the comparable messenger levels of “Imari” vs the smooth cultivars may subtend the low content differences in this STL class (Table 6). The *83192/GAS* / expression recurred as more abundant in curly than smooth cultivars (Fig. 4c, Fig. 7), supporting a conserved role to determine higher amounts of lactucopicrin. A significant positive correlation of this *GAS* gene was found with a downstream *GAO* gene (*11533/GAO*), whereas no associated *COS* genes were identified. This may be due to a limited level of transcriptome functional annotation, caused by the fragmentary knowledge on the STL pathway that prevented the widening of *COS* gene pool. The identification of novel GAS and GAO putative enzymes involved in lactucopicrin biosynthesis may turn a relevant information, considering that they appear to be phylogenetically near a lettuce GAS cluster with uncharacterized function. Indeed, a three-way significant positive correlation is supportive for the control of *83192/GAS* by the *86458/WRKY* and *72724/MYB* TFs in Lp synthesis. Moreover, promoter sequence analysis of lettuce *GAS* gene 90% identical to endive 83192/*GAS* (Table S9) scored numerous target motifs for WRKY and MYB-like factors (Table S14). Inherently, the endive deduced proteins of *32240* and *32243/MYB_related* genes were 65% identical to Arabidopsis LHY-CCA1-LIKE1 TFs (Table S13) that are co-expressed and highly correlated to several isoprenoid genes in photosynthetic tissues^63^. Moreover, a few WRKY factors can control sesquiterpene biosynthesis^64^; the endive 86458/WRKY shares identity with the Arabidopsis WRKY70, an upstream effector of MYC2 factor^65^ that regulates transcription of two terpene synthases^66^. Consequently, the presence of putative WRKY and MYB binding sites in the promoter of the *L. sativa* homologous of *83192/GAS* gene may imply that the latter is also a direct target in endive. The strong positive correlation of both *86458/WRKY* and *72724/MYB* vs Lc and DHLc contents, which significantly grouped with Lp (Fig. 6), suggests that the branch leading to Lp, Lc, and DHLc might be under a common regulatory network. The negative correlation of *32240* and *32243/MYB_related* gene expression vs those of *86458/WRKY* and *72724/MYB* and the amounts of Lp, Lc, and DHLc reinforces the latter hypothesis. The transcription of all identified *TF* did not show significant correlation with dLc/DHdLc/DHLp levels, leading to hypothesize the existence of two separated routes in the synthesis of Lp/Lc/DHLc and DLc/DHdLc/DHLp. Moreover, different *GAS/GAO* genes correlated with the two STL group types, which further supports the likeliness of a branching point at the germacrene synthase level before costunolide formation.

Looking at the STLTOT contents, ranges were higher than those found in a survey (128–264 and 235–2045 mg/kg d.m. in curly and smooth types respectively) based on 32 accessions^12^, but, consistently, the average content of curly genotypes was maintained over 2.4-fold higher than escaroles. The relative levels of LcTOT and LpTOT sub-classes (out of total STL) were 64.9% and 35.1% in curly endives similarly to those of escaroles (62.7% and 37.2%). These values differed from other results^20^ reporting that LcTOT and LpTOT ratios were 77% and 22% in curly vs 82% and 16 % in smooth endives. Several factors may be evoked to explain these discrepancies including diversity of cultivars, leaf types and their positon in the rosette, cultivation and environmental conditions, which influence STL leaf content and composition of endive-related species such as chicory and lettuce^67,68^. Although sensory analyses were beyond the scope of this work, predicted bitterness based on STL perception thresholds^18^ distinguished curly from smooth types, consistently with taste based bitterness indices of endive^20^. Moreover, Lc, DHLc, and Lp were the best discriminants of curly vs escarole types (respectively 3.7-, 2.1-, and 2.3-fold higher in the former). The Lp content sensibly altered predicted bitterness due to its much lower index than Lc and DHLc (0.5 vs 1.6–1.7 p.p.m.), and was consistent with the Lp dominant effect measured in sensory tests on endive^19^.

In conclusion, the assembled transcriptome was effective to assess differences between curly- and smooth-leafed cultivars at both the allelic and gene expression levels, and useful to characterize the STL biosynthesis pathway in endive. Specifically, a set of *GAS/GAO/COS* genes with coordinated/correlated expression to the contents of DLc/DHdLc/DHLp was identified and the specific relationship among *86458/WRKY*, *72724/MYB*, and *83192/GAS* was inferred for the Lp/Lc/DHLc branch. These findings open perspectives for further investigating these two key branches, considering that genes downstream the *BS* module have been unknown so far, as well as those of catabolism and transport.

## Electronic supplementary material

Supplementary Tables S1-S4

Supplementary Tables S6-S14

Supplementary Figures 1–6

Supplementary Table S5
