# Supplementary material for: Transcriptome driven characterization of curly- and smooth-leafed endives reveals molecular differences in the sesquiterpenoid pathway
Source: Hortic Res. 2019 Jan 1;6:1. doi: 10.1038/s41438-018-0066-6 (PMC6312536; doi:10.1038/s41438-018-0066-6)
Supplement: Supplementary file 1 — Supplementary Tables S1-S4 [file 41438_2018_66_MOESM1_ESM.docx]

# Supplementary tables S1-S4

### Table S1. Major phenotypical parameters of endives and escaroles.

| **Cultivar** | **Head weight (gr)** | **Targeted leaves** | | | |
| --- | --- | --- | --- | --- | --- |
|  |  | **Fresh weight (gr)** | **dw/fw (%)** | **Length (cm)** | **Surface (cm^2^)** |
| Domari | 721.0±19.4 | 95.2±19.4 | 5.2±0.7 | 25.0±2.4b | 124.0±13.9ab |
| Imari | 767.0±160.1 | 104.4±28.1 | 5.3±0.7 | 23.3±1.4c | 110.4±17.1b |
| Myrna | 872.4±189.2 | 79.0±11.0 | 5.4±1.5 | 28.8±2.5a | 76.8±19.1c |
| Confiance | 721.8±166.1 | 76.9±21.1 | 5.1±0.8 | 17.2±1.2e | 117.7±35.7b |
| Flester | 841.3±191.1 | 100.5±19.7 | 4.8±1.4 | 20.1±1.3d | 145.7±21.8a |
| *Significance* | *n.s.* | *n.s.* | *n.s.* | **** | ***** |

Data are presented as means±standard deviation. 15 heads per cultivar were weighed; 15 leaves (5 leaves per 3 distinct plants for each cultivar) were photographed and weighed. The “digimizer” software (www.digimizer.com) was used to compute surface and length. Means marked with the same letters were not significantly differ after the ANOVA and HSD Tukey’s test. N.s., non-significant. **, *** indicate significance at P < 0.01 and 0.001, respectively.

### Table S2. Primer list

| **Unigene** | **Nr annotation** | **Primers (5'>3')** | **Analysis** |
| --- | --- | --- | --- |
| Ce_contig5542 | *AP2/ERF domain-containing protein* | Fw: GGGAGCGGTGGTAGTAATGA  Bw: TTTCTGTTTGGCTCGTCGTC | HRM |
| Ce_contig5542 | *AP2/ERF domain-containing protein* | Fw: ATCCCACCACCAACATCGAA Bw: TGAAACCTTCGTCAATGGCG | HRM |
| Ce_contig16955 | *Germacrene A synthase long form* | Fw: TGGTTTGATTACTTCGGCTTA Bw: GAGCCTCTTCGTCTGCAATC | HRM |
| Ce_contig16955 | *Germacrene A synthase long form* | Fw: CTACGGGTGTGGATGCCTAT Bw: TTTCCATGCGTTTTCAATCA | HRM |
| Ce_contig17454 | *CO dehydrogenase flavoprotein-like,*  *FAD-binding, subdomain 2* | Fw: TCTAGCCTCTACCCTGACCC Bw: TGATTTGACAACGCTTCCCT | HRM |
| Ce_contig22657 | *CheY-like superfamily* | Fw: ACAAGGCCAGCAGTTCAAAC Bw: GGGTACTGAGTTGACCCGAA | HRM |
| Ce_contig32702 | *Chase-Like Protein* | Fw: CTGTGCTCTCACAGGACCAA Bw: ACGAAGATGTCCTTGATGGG | HRM |
| Ce_contig46826 | *Emopamil-Binding, Partial* | Fw: GATCCGATCATGGAGGAAGA Bw: ATCATGCAAACACTCCCACA | HRM |
|  |  |  |  |
| Ce_contig5291 | *Inositol polyphosphate kinase* | Fw: TTCTTGGGTGGGCTTTGTTC Bw: GCTGTCTGTGGCTCAATCTG | HRM |
| Ce_contig84215 | *Cation-Transporting P-Type Atpase* | Fw: TCCACACAATCACGGCTAGT  Bw: CCACAACACTTCTTCCTGCA | HRM |
| Ce_contig3930 | *UDP-glucuronosyl/UDP-glucosyltransferase* | Fw: AGAGAGCAATGGAGGAAGGG Bw: CGCGGATGTTACACCCATTT | HRM |
| Ce_contig32501 | *Metallophosphoesterase Domain-Containing* | Fw: GTCTGACCCTGATGACCGTT Bw: TGAAACCCTCCATAACCAGCT | HRM |
| Ce_contig23588 | *Rac-like GTP-binding protein ARAC1* | Fw: GAAAGCGGGTCAAAAGGCTT Bw: GCCTCGATGACTTTGACTTCC | HRM |
| Ce_contig80048 | *polyphenol oxidase* | Fw: TGCAAATCCGATCATGGCAC Bw: TCTTTGTGGTGGATGGAGGG | HRM |
| Ce_contig43315 | *Pyruvate Kinase* | Fw: TGGAATCAATCGCTTCATCCG  Bw: CAATCAACCTTGCAGCCCTT | HRM |
| Ce_contig16955 | *Germacrene A synthase* | Fw: GATTTCAAGGCTCCAAGACG | qPCR |
|  |  | Bw: TCTTGGATCGCCACTTCTTC |  |
| Ce_contig41447 | *Germacrene A Synthase* | Fw: TGGGCTGCCAGAAAATATAGC | qPCR |
|  |  | Bw: GAGACGCGACCAATCACG |  |
| Ce_contig52991 | *Germacrene A Synthase* | Fw: ACAGTGGATACGTGGCATCA | qPCR |
|  |  | Bw: TCGCCCATACCCACTAAAGC |  |
| Ce_contig81731 | *Germacrene A Synthase* | Fw: CGGAGTATCAGAAGATGTAGCTG | qPCR |
|  |  | Bw: GCCAGCAATTCTATGGAAACC |  |
| Ce_contig83192 | *Germacrene A Synthase* | Fw: CATGGAGGAGCCAAAAGAAG | qPCR |
|  |  | Bw: CTCTTCCGCGAAATGGTATC |  |
| Ce_contig46043 | *Germacrene A Oxidase* | Fw: ATCGCCATTTCTCCACACTC | qPCR |
|  |  | Bw: CCCATTTTGGAGATGACACC |  |
| Ce_contig84591 | *Costunolide Synthase* | Fw: ACCGGCAAGAGCATCATAAC | qPCR |
|  |  | Bw: TAAAACCCCATGAGCTCGAC |  |
| Ce_contig82792 | *β-Caryophyllene Synthase* | Fw: TTGGGGAGACCAGTTTCTTG | qPCR |
|  |  | Bw: GCAAGAGTCGAAACCAAAGG |  |

### Table S3. Unigene annotation overview.

| **Databases** | **Unigene n.** | **Unigene %** |
| --- | --- | --- |
| Nr | 56,246 | 73.0 |
| TrEMBL | 56,976 | 74,0 |
| RefSeq | 54,516 | 70.8 |
| Tair | 51,697 | 67.1 |
| SwissProt | 37,873 | 49.2 |
| InterPro | 33,278 | 43.2 |
| GO | 40,626 | 52.7 |
| KEGG | 15,430 | 20.0 |
| KOG | 11,785 | 15.3 |
| PlantTFdb | 5,475 | 7.1 |

### Table S4. Distribution of percent length coverage bins for the best matching proteins against Nr database entries.

| Length coverage range (%) | Bin | Count in bin | Cumulative count |
| --- | --- | --- | --- |
| 90-100 | 100 | 17,002 | 17,002 (30%) |
| 80-90 | 90 | 3,733 | 20,735 (37%) |
| 70-80 | 80 | 3,417 | 24,152 (43%) |
| 60-70 | 70 | 3,767 | 27,919 (50%) |
| 50-60 | 60 | 4,229 | 32,148 (57%) |
| 40-50 | 50 | 4,452 | 36,600 (65%) |
| 30-40 | 40 | 4,941 | 41,541 (74%) |
| 20-30 | 30 | 5,508 | 47,049 (84%) |
| 10-20 | 20 | 5,983 | 53,032 (94%) |
| 0-10 | 10 | 3,214 | 56,246 (100%) |
