# Supplementary material for: Transcriptome driven characterization of curly- and smooth-leafed endives reveals molecular differences in the sesquiterpenoid pathway
Source: Hortic Res. 2019 Jan 1;6:1. doi: 10.1038/s41438-018-0066-6 (PMC6312536; doi:10.1038/s41438-018-0066-6)
Supplement: Supplementary file 2 — Supplementary Tables S6-S14 [file 41438_2018_66_MOESM2_ESM.docx]

# Supplementary tables S6-S13

### Table S6. SSR mining statistics.

| Parameters | Values |
| --- | --- |
| Total number of sequences examined: | 77,022 |
| Total size of examined sequences (bp): | 96,091,363 |
| Total number of identified SSRs: | 19,951 |
| Number of sequences containing SSRs: | 15,940 |
| Number of sequences containing more than 1 SSR: | 3,155 |
| Number of SSRs present in compound formation: | 1,184 |

### Table S7. GO and KEGG enrichment analyses of unigenes bearing core-SNPs ^1^

| **Description** | **Annotations^2^** | **Unigenes^3^** | **Background^4^** | **FDR^5^** |
| --- | --- | --- | --- | --- |
| Membrane | C:GO:0016020 | 37 | 1,489 | 4.31E-02 |
| Hydrolase activity | F:GO:0016787 | 34 | 1,378 | 4.63E-02 |
| Proteolysis | P:GO:0006508 | 29 | 904 | 3.83E-02 |
| Transferase activity, transferring P-containing groups | F:GO:0016772 | 24 | 904 | 4.45E-02 |
| Transferase activity, transferring hexosyl groups | F:GO:0016758 | 12 | 314 | 3.83E-02 |
| Transaminase activity | F:GO:0008483 | 7 | 136 | 3.83E-02 |
| mRNA splicing, via spliceosome | P:GO:0000398 | 5 | 107 | 4.71E-02 |
| Acetylglucosaminyltransferase activity | F:GO:0008375 | 5 | 71 | 3.83E-02 |
| Lipid storage | P:GO:0019915 | 4 | 33 | 3.83E-02 |
| Photomorphogenesis | P:GO:0009640 | 4 | 61 | 4.31E-02 |
| RNA degradation | KEGG map03018 | 8 | 201 | 3.65E-02 |
| Fructose and mannose metabolism | KEGG map00051 | 5 | 59 | 1.81E-02 |
| Valine leucine and isoleucine biosynthesis | KEGG map00290 | 3 | 26 | 2.35E-02 |

**1**, The table includes GO and KEGG pathways with at least three genes. GO annotation comprised 40,626 unigenes and 735 bore core-SNPs. KEGG annotation comprised 15,431 unigenes and 284 bore core-SNPs. **2**, GO (P, biological process; C, cellular compartment; F, molecular function) and KEGG annotation codes. **3**, number of unigenes with core-SNPs within each specific GO term or KEGG map. **4**, number of unigenes in the transcriptome within each specific GO term or KEGG map. **5**, false discovery rate (P≤0.05).

### Table S8. Validation of SNPs in *C. endivia* cultivars

|  |  | **SNP by RNA-seq** | | | | | | | | | | **SNP validation by HRM** | | | | |
| --- | --- | --- | --- | --- | --- | --- | --- | --- | --- | --- | --- | --- | --- | --- | --- | --- |
|  |  | **Domari** | | **Imari** | | **Myrna** | | **Confiance** | | **Flester** | | **Domari** | **Imari** | **Myrna** | **Confiance** | **Flester** |
| **Unigene** | POS^1^ | REF^2^ | Gt^3^ | Al^4^ | Gt | Al | Gt | Al | Gt | Al | Gt | Gt | Gt | Gt | Gt | Gt |
| Ce_contig5542 | 796 | A | *wt* | - | *wt* | G | *ho* | G | *ho* | G | *ho* | *wt* | *wt* | *ho* | *ho* | *ho* |
| Ce_contig5542 | 1353 | A | *wt* | G | *ho* | G | *ho* | G | *ho* | G | *ho* | *wt* | *wt* | *ho* | *ho* | *ho* |
| Ce_contig16955 | 1419 | T | *wt* | A | *ho* | - | *wt* | - | *wt* | - | *wt* | *wt* | *ho* | *wt* | *ho** | *wt* |
| Ce_contig16955 | 1621 | C | *wt* | A | *ho* | - | *wt* | - | *wt* | - | *wt* | *wt* | *ho* | *wt* | *wt* | *wt* |
| Ce_contig17454 | 1735 | A | *wt* | - | *wt* | - | *wt* | C | *ho* | - | *wt* | *wt* | *wt* | *wt* | *ho* | *wt* |
| Ce_contig22657 | 1191 | T | *wt* | C | *ho* | C | *ho* | C | *ho* | C | *ho* | *wt* | *ho* | *ho* | *ho* | *ho* |
| Ce_contig32702 | 922 | C | *wt* | - | *wt* | A | *he* | A | *ho* | A | *ho* | *wt* | *wt* | *he* | *ho* | *ho* |
| Ce_contig46826 | 721 | C | *wt* | - | *wt* | - | *wt* | - | *wt* | T | *ho* | *wt* | *wt* | *wt* | *wt* | *ho* |
| Ce_contig46826 | 772 | T | *wt* | - | *wt* | - | *wt* | - | *wt* | G | *ho* | *wt* | *wt* | *wt* | *wt* | *ho* |
| Ce_contig5291 | 1572 | C | *wt* | - | *wt* | - | *wt* | - | *wt* | T | *ho* | *wt* | *wt* | *wt* | *wt* | *ho* |
| Ce_contig84215 | 1054 | T | *wt* | - | *wt* | - | *wt* | C | *ho* | - | *wt* | *wt* | *wt* | *wt* | *ho* | *wt* |
| Ce_contig3930 | 1617 | C | *wt* | - | *wt* | - | *wt* | - | *wt* | T | *ho* | *wt* | *wt* | *wt* | *ho** | *ho* |
| Ce_contig32501 | 964 | T | *wt* | - | *wt* | - | *wt* | C | *ho* | - | *wt* | *wt* | *wt* | *wt* | *ho* | *wt* |
| Ce_contig23588 | 988 | G | *wt* | T | *ho* | - | *wt* | T | *ho* | T | *ho* | *wt* | *ho* | *wt* | *ho* | *ho* |
| Ce_contig80048 | 323 | A | *wt* | - | *wt* | - | *wt* | G | *ho* | - | *wt* | *wt* | *wt* | *wt* | *ho* | *wt* |
| Ce_contig43315 | 1416 | C | *wt* | - | *wt* | - | *wt* | T | *ho* | T | *ho* | *wt* | *wt* | *wt* | *ho* | *ho* |

**1**, position of the nucleotide point mutation along the contig sequence; **2**, base of the Domari reference sequence; **3**, genotype; *wt*, reference base in homozygous state; *ho* and *he*, alternative base in homo- and heterozygous state, respectively. *, discrepancies between prediction and validation. **4**, alternative base.

### Table S9. Costunolide branch of endive STL unigenes *vs* *Lactuca sativa* genome.

|  | *C. endivia* | | *Lactuca sativa* early genome | | | | | |
| --- | --- | --- | --- | --- | --- | --- | --- | --- |
|  | **Query**^1^ | **FL%**^2^ | **Peptide name** | **id%** | **Evalue** | **Score** | **Description** | **EC** |
| *GAS* | 41447 | 100 | Lsat_1_v5_gn_1_123521.2 | 91.1 | 0.0E+00 | 980 | Germacrene-A synthase | 4.2.3.23 |
|  | 83192 | 100 | Lsat_1_v5_gn_1_123521.1 | 90.2 | 0.0E+00 | 973 | Germacrene-A synthase | 4.2.3.23 |
|  | 52991 | 100 | Lsat_1_v5_gn_8_116421.1 | 95.0 | 0.0E+00 | 1040 | Germacrene-A synthase | 4.2.3.23 |
|  | 16955 | 97 | Lsat_1_v5_gn_8_116421.3 | 86.6 | 0.0E+00 | 860 | Germacrene-A synthase | 4.2.3.23 |
|  | 81731 | 64 | Lsat_1_v5_gn_1_123521.2 | 81.6 | 3.0E-178 | 503 | Germacrene-A synthase | 4.2.3.23 |
| *GAO* | 46043 | 98 | Lsat_1_v5_gn_8_115261.3 | 94.1 | 0.0E+00 | 1039 | Germacrene A-alcohol dehydrogenase/ -hydroxylase | 1.14.13.123; 1.1.1.314 |
|  | **47698** | 92 | Lsat_1_v5_gn_1_123721.1 | 73.5 | 3.0E-161 | 468 | Germacrene A-alcohol dehydrogenase/ -hydroxylase | 1.14.13.123; 1.1.1.314 |
|  | **11533** | 92 | Lsat_1_v5_gn_3_65300.1 | 72.4 | 9.0E-165 | 476 | Germacrene A-alcohol dehydrogenase/ -hydroxylase | 1.14.13.123; 1.1.1.314 |
| *COS* | 84591 | 100 | Lsat_1_v5_gn_7_33721.1 | 98.6 | 0.0E+00 | 918 | Costunolide synthase | 1.14.13.120 |
|  | **69070** | 96 | Lsat_1_v5_gn_5_60700.1 | 89.9 | 0.0E+00 | 753 | Costunolide synthase | 1.14.13.120 |
|  | **34331** | 75 | Lsat_1_v5_gn_5_60700.1 | 90.0 | 0.0E+00 | 610 | Costunolide synthase | 1.14.13.120 |

**1**, Unigene numbers. The sequences identified as new putative *GAO* and *COS* after BlastX analysis on *L. sativa* genome are bolded. **2**, FL, Full-length coverage with respect to the *L. sativa* deduced proteins.

### Table S10. SNP analysis in the STL unigenes

|  |  |  |  | **Curly-leafed endives** | | | | | | | | | | | | | | | | | | **Ecaroles** | | | | | | | | | | | |
| --- | --- | --- | --- | --- | --- | --- | --- | --- | --- | --- | --- | --- | --- | --- | --- | --- | --- | --- | --- | --- | --- | --- | --- | --- | --- | --- | --- | --- | --- | --- | --- | --- | --- |
|  |  |  |  | **Domari** | | | | | | **Imari** | | | | | | **Myrna** | | | | | | **Confiance** | | | | | | **Flester** | | | | | |
| **Unigene** | **Annot^1^** | **POS^2^** | **REF^3^** | **Al^4^** | **Qu^5^** | **To^6^** | **Gt^7^** | **Gq^8^** | **Type^9^** | **Al** | **Qu** | **To** | **Gt** | **Gq** | **Type** | **Al** | **Qu** | **To** | **Gt** | **Gq** | **Type** | **Al** | **Qu** | **To** | **Gt** | **Gq** | **Type** | **Al** | **Qu** | **To** | **Gt** | **Gq** | **Type** |
| 16955 | GAS | 192 | C | . | . | . | wt | . | . | T | 222 | 47 | ho | 99 | Si | . | . | . | wt | . | . | . | . | . | wt | . | . | . | . | . | wt | . | . |
|  |  | 693 | A | . | . | . | wt | . | . | G | 222 | 68 | ho | 99 | Si | . | . | . | wt | . | . | . | . | . | wt | . | . | . | . | . | wt | . | . |
|  |  | 783 | C | . | . | . | wt | . | . | T | 222 | 80 | ho | 99 | Si | . | . | . | wt | . | . | . | . | . | wt | . | . | . | . | . | wt | . | . |
|  |  | 855 | A | . | . | . | wt | . | . | G | 222 | 79 | ho | 99 | Si | . | . | . | wt | . | . | . | . | . | wt | . | . | . | . | . | wt | . | . |
|  |  | 1023 | C | . | . | . | wt | . | . | T | 222 | 65 | ho | 99 | Si | . | . | . | wt | . | . | . | . | . | wt | . | . | . | . | . | wt | . | . |
|  |  | 1221 | G | . | . | . | wt | . | . | A | 222 | 84 | ho | 99 | Si | . | . | . | wt | . | . | . | . | . | wt | . | . | . | . | . | wt | . | . |
|  |  | 1281 | A | . | . | . | wt | . | . | G | 222 | 56 | ho | 99 | Si | . | . | . | wt | . | . | . | . | . | wt | . | . | . | . | . | wt | . | . |
|  |  | 1326 | G | . | . | . | wt | . | . | A | 222 | 53 | ho | 99 | Si | . | . | . | wt | . | . | . | . | . | wt | . | . | . | . | . | wt | . | . |
|  |  | **1419** | **T** | . | . | . | wt | . | . | **A** | **222** | **80** | ho | **99** | **Si** | . | . | . | wt | . | . | . | . | . | wt | . | . | . | . | . | wt | . | . |
|  |  | 1560 | A | . | . | . | wt | . | . | G | 222 | 79 | ho | 99 | Si | . | . | . | wt | . | . | . | . | . | wt | . | . | . | . | . | wt | . | . |
|  |  | **1621** | **C** | . | . | . | wt | . | . | **A** | **222** | **79** | ho | **99** | **Ms (Q>K)** | . | . | . | wt | . | . | . | . | . | wt | . | . | . | . | . | wt | . | . |
| 52991 | GAS | 639 | G | . | . | . | wt | . | . | . | . | . | wt | . | . | . | . | . | wt | . | . | A | 56 | 14 | he | 82 | Ms (A>T) | . | . | . | wt | . | . |
|  |  | 642 | A | . | . | . | wt | . | . | . | . | . | wt | . | . | C | 46 | 32 | he | 79 | Ms (K>Q) | C | 66 | 16 | he | 76 | Ms (K>Q) | C | 58 | 20 | he | 91 | Ms (K>Q) |
|  |  | 662 | C | G | 114 | 27 | he | 99 | Ms (N>L) | G | 46 | 14 | he | 79 | Ms (N>L) | G | 33 | 40 | he | 66 | Ms (N>L) | G | 82 | 26 | he | 99 | Ms (N>L) | G | 117 | 30 | he | 99 | Ms (N>L) |
|  |  | 707 | A | G | 54 | 31 | he | 87 | Si | . | . | . | wt | . | . | G | 56 | 44 | he | 89 | Si | . | . | . | wt | . | . | G | 84 | 25 | he | 99 | Si |
|  |  | 773 | T | . | . | . | wt | . | . | . | . | . | wt | . | . | . | . | . | wt | . | . | A | 34 | 14 | he | 67 | Ms (D>E) | . | . | . | wt | . | . |
|  |  | 779 | T | C | 49 | 25 | he | 82 | Si | . | . | . | wt | . | . | C | 34 | 31 | he | 67 | Si | C | 51 | 15 | he | 84 | Si | C | 70 | 28 | he | 99 | Si |
|  |  | 827 | A | G | 38 | 26 | he | 71 | Si | . | . | . | wt | . | . | . | . | . | wt | . | . | . | . | . | wt | . | . | G | 43 | 29 | he | 76 | Si |
|  |  | 828 | G | C | 37 | 26 | he | 70 | Ms (E>Q) | . | . | . | wt | . | . | . | . | . | wt | . | . | . | . | . | wt | . | . | C | 43 | 29 | he | 76 | Ms (E>Q) |
|  |  | 857 | T | A | 70 | 35 | he | 99 | Si | . | . | . | wt | . | . | A | 85 | 52 | he | 99 | Si | A | 50 | 37 | he | 83 | Si | A | 37 | 37 | he | 70 | Si |
| 46043 | GAO | 608 | T | . | . | . | wt | . | . | . | . | . | wt | . | . | . | . | . | wt | . | . | . | . | . | wt | . | . | C | 45 | 84 | he | 78 | Si |
| 69070 | COS | 204 | T | C | 52 | 15 | he | 85 | Si | . | . | . | wt | . | . | . | . | . | wt | . | . | . | . | . | wt | . | . | . | . | . | wt | . | . |
|  |  | 208 | G | T | 39 | 19 | he | 72 | Ms (A>S) | . | . | . | wt | . | . | . | . | . | wt | . | . | . | . | . | wt | . | . | . | . | . | wt | . | . |
|  |  | 252 | A | C | 57 | 15 | he | 87 | Si | . | . | . | wt | . | . | . | . | . | wt | . | . | . | . | . | wt | . | . | . | . | . | wt | . | . |
|  |  | 262 | A | . | . | . | . | . | . | . | . | . | wt | . | . | G | 41 | 24 | wt | 74 | Ms (I>V) | . | . | . | wt | . | . | G | 48 | 17 | wt | 81 | Ms (I>V) |
|  |  | 310 | C | T | 81 | 19 | he | 99 | Si | . | . | . | wt | . | . | . | . | . | wt | . | . | . | . | . | wt | . | . | . | . | . | wt | . | . |
| 84591 | COS | 340 | A | C | 57 | 68 | he | 90 | Si | . | . | . | wt | . | . | . | . | . | wt | . | . | . | . | . | wt | . | . | . | . | . | wt | . | . |
|  |  | 393 | A | . | . | . | wt | . | . | G | 71 | 57 | he | 99 | Ms (N>S) | . | . | . | wt | . | . | . | . | . | wt | . | . | . | . | . | wt | . | . |
|  |  | 402 | A | . | . | . | wt | . | . | G | 48 | 58 | he | 81 | Ms (K>R) | . | . | . | wt | . | . | . | . | . | wt | . | . | . | . | . | wt | . | . |
|  |  | 490 | G | A | 31 | 76 | he | 64 | Si | . | . | . | wt | . | . | . | . | . | wt | . | . | . | . | . | wt | . | . | . | . | . | wt | . | . |
|  |  | 496 | C | T | 48 | 74 | he | 81 | Si | . | . | . | wt | . | . | . | . | . | wt | . | . | . | . | . | wt | . | . | . | . | . | wt | . | . |
|  |  | 775 | G | A | 55 | 74 | he | 88 | Si | . | . | . | wt | . | . | . | . | . | wt | . | . | . | . | . | wt | . | . | . | . | . | wt | . | . |
|  |  | 1348 | C | T | 68 | 64 | he | 99 | Si | . | . | . | wt | . | . | . | . | . | wt | . | . | T | 56 | 69 | he | 89 | Si | . | . | . | wt | . | . |

**1**, Annotation symbol. GAS, Germacrene A synthase; GAO, Germacrene A oxidase; COS, Costunolide synthase. **2**, Position of the nucleotide point mutation along the contig sequence. **3**, Base of the Domari reference sequence. **4**, Alternative base. **5**, Phred-scaled quality score for the assertion made in ALT. i.e. -10log_10_prob (alternative base call is wrong). **6**, Total number of high-quality reference and alternative bases (in both forward and reverse orientation) used in variant calling. **7**, Genotype; wt, reference base in homozygous state; ho and he, alternative bases in homo- and heterozygous state, respectively. **8**, Genotype quality. Phred quality -10log_10_prob(genotype call is wrong). **9**, Substitution type. As for missense substitutions (Ms), the amino acid changes were bracketed. Si, silent substitutions. Private SNPs are grey-shaded whilst SNPs validated by HRM were bolded.

### Table S11. Deduced bitterness in leaves of curly and smooth endives

|  | **Bitterness scores^1^** | | | | | | | | |
| --- | --- | --- | --- | --- | --- | --- | --- | --- | --- |
| **Cultivars** | **Lc** | **DHLc** | **dLc** | **DHdLc** | **LcTOT** | **Lp** | **DHLp** | **LpTOT** | **STLTOT** |
| 'Domari' | 341.3±10.3b | 210.9±9.4a | 140.3±9.5c | 319.9±15.3b | 1012.4±12.3b | 824.6±36.6c | 712±50.3a | 1536.6±84.8b | 2549±89.8b |
| 'Imari' | 279.1±9.8c | 147.5±10.5b | 89.5±7.8d | 73.1±3.9e | 589.3±28.7c | 1953.8±43.2a | 5.0±5.0e | 1958.8±38.6a | 2548.1±14.6b |
| 'Myrna' | 408.9±10a | 128.9±10.9b | 759.4±17.1a | 370.7±14.2a | 1668±30.7a | 1506.2±40.8b | 367±22.5b | 1873.2±44.5a | 3541.2±69.2a |
| 'Confiance' | 90.9±6.6d | 70.1±7.6c | 174.1±12.9b | 183.7±11c | 518.8±10.8d | 800.6±30.8c | 245.5±31.5c | 1046.1±9.9c | 1564.9±9.4c |
| 'Flester' | 94.4±6.4d | 80.8±8.1c | 91.8±9.5d | 133.9±10.5d | 400.9±34.4e | 429.2±28.2d | 146.5±6d | 575.7±34.2d | 976.6±10.3d |
| *Significance* | ***** | ***** | ***** | ***** | ***** | ***** | ***** | ***** | ***** |
| Curly-type | 343.1±56.9a | 162.5±38.3a | 329.7±323.1 | 254.6±138.3 | 1089.9±471.2a | 1428.2±493.7a | 361.3±307.4 | 1789.5±200.1a | 2879.4±499.6a |
| Smooth-type | 92.6±6.1b | 75.5±9.1b | 133.0±46.2 | 158.8±28.9 | 459.9±68.5b | 614.9±205.1b | 196.0±57.9 | 810.9±258.6b | 1270.8±322.4b |
| *Significance* | ***** | ***** | *n.s.* | *n.s.* | **** | **** | *n.s.* | ***** | ***** |

**1**, Calculated as ratio between STL amounts and the bitter threshold in water as measured by Van Beek *et al.* (1990) and reported as mean values ± standard deviation. Lc, lactucin; DHLc, 11(S),13-dihydrolactucin; dLc, 8-deoxylactucin; DHdLc, 11(S),13-dihydro-8-deoxylactucin; Lp, lactucopicrin; DHLp, 11(s),13-dihydrolactucopicrin; LcTOT, total lactucin-like STLs; LpTOT, total lactucopicrin-like STLs. n.s., non-significant.*, **, *** = significant at P < 0.05, 0.01 and 0.001, respectively after after the ANOVA and HSD Tukey’s test.

### Table S12. *C. endivia* transcription factors annotation.

| ***C. endivia*** | | | **PlantTFdb** | | | | **nr NCBI (*Lactuca sativa*)** | | | | **TAIR10** | | | |
| --- | --- | --- | --- | --- | --- | --- | --- | --- | --- | --- | --- | --- | --- | --- |
| **Query**^1^ | **FL%**^2^ | **aa** | **Hit ID**^3^ | **TF family** | **id%** | **Evalue** | **Hit ID** | **Description** | **id%** | **Evalue** | **Hit ID** | **Description** | **id%** | **Evalue** |
| 32240 | 100 | 288 | Lsa014527 | MYB_related | 82.7 | 1e-121 | XP_023767856.1 | RVE8/ LHY-CCA1-LIKE1 | 89.0 | 4e-171 | AT5G02840.3 | LHY/CCA1-LIKE1 | 66.6 | 2e-87 |
| 32243 | 100 | 263 | Lsa014527 | MYB_related | 88.7 | 1e-151 | XP_023767857.1 | RVE8/ LHY-CCA1-LIKE1 | 88.7 | 3e-155 | AT5G02840.3 | LHY/CCA1-LIKE1 | 67.3 | 1e-85 |
| 72724 | 88 | 536 | Achn077281 | MYB_related | 63.3 | 8e-51 | XP_023743528.1 | Polyubiquitin | 67.3 | 0e+00 | AT4G05320.4 | UBQ10 | 67.3 | 1e-157 |
| 74591 | 100 | 504 | MDP0000173550 | MYB | 31.3 | 7e-45 | XP_023744282.1 | TTF-type Zf | 53.4 | 7e-146 | AT1G19260.1 | TTF-type Zf | 47.2 | 1e-115 |
| 86458 | 100 | 279 | Lsa005482 | WRKY | 76.2 | 1e-151 | XP_023737392.1 | WRKY70 | 83.1 | 3e-154 | AT3G56400 | WRKY70 | 43.4 | 4e-38 |

**1**, Unigene numbers. **2**, FL, Full-length coverage with respect to the *L. sativa* deduced proteins. **3**, Lsa, *Lactuca sativa*; Achn, *Actinidia chinensis*; MD, *Malus domestica.*

### Table S13. Differential biosynthesis gene expression in the curly *vs* smooth cultivar comparisons.

| **Annotation** | **Unigenes** | **Size**  **(bp)** | **DvsC** | | **DvsF** | | **IvsC** | | **IvsF** | | **MvsC** | | **MvsF** | |
| --- | --- | --- | --- | --- | --- | --- | --- | --- | --- | --- | --- | --- | --- | --- |
|  |  |  | **log_2_FC** | **FDR** | **log_2_FC** | **FDR** | **log_2_FC** | **FDR** | **log_2_FC** | **FDR** | **log_2_FC** | **FDR** | **log_2_FC** | **FDR** |
| *GAS* | 16955 | 2898 | 0.25 | n.s. | -0.34 | n.s. | 0.30 | n.s. | -0.33 | n.s. | 0.72 | * | 0.12 | n.s. |
|  | 41447 | 1874 | 0.42 | n.s. | 1.21 | *** | -0.44 | n.s. | 0.32 | n.s. | 1.75 | *** | 2.53 | *** |
|  | 52991 | 1903 | 1.46 | * | 0.62 | n.s. | 0.35 | n.s. | -0.53 | n.s. | 2.96 | *** | 2.11 | *** |
|  | **83192** | **1901** | **1.66** | ****** | **4.40** | ******* | **2.43** | ******* | **5.77** | ******* | **1.78** | ****** | **4.16** | ******* |
| *GAO* | 46043 | 2064 | 0.42 | n.s. | -0.04 | n.s. | -0.15 | n.s. | -0.65 | n.s. | 0.73 | n.s. | 0.25 | n.s. |
|  | 47698 | 1887 | 0.50 | n.s. | 0.94 | ** | 0.37 | n.s. | 0.77 | n.s. | -0.23 | n.s. | 0.17 | n.s. |
|  | 11533 | 1787 | -0.31 | n.s. | 2.21 | *** | -0.17 | n.s. | 2.33 | *** | -0.12 | n.s. | 2.41 | *** |
| *COS* | 84591 | 1984 | 0.33 | n.s. | -0.20 | n.s. | -0.65 | n.s. | -1.22 | *** | 0.85 | * | 0.31 | n.s. |
|  | 69070 | 1693 | 0.39 | n.s. | -0.04 | n.s. | -0.76 | n.s. | -1.23 | ** | 1.69 | *** | 1.25 | *** |

Up- and down-regulated genes are green- and red-shaded, respectively; Core-DEG are bolded; novel *GAO* and *COS* identified by blasting lettuce genome are in underlined; D, 'Domari'; I, 'Imari'; M, 'Myrna'; C, 'Confiance'; F, 'Flester'. *, **, *** = significant at P ≤ 0.05, 0.01 and 0.001, respectively; n.s., non-significant. *GAS*, Germacrene A synthase; *GAO,* Germacrene A oxidase; *COS,* Costunolide synthase.

### Table S14. Promoter analysis of the lettuce *GAS* orthologue to endive contig83192

| **Binding site** | **Name** | **Signal Sequence** | **bp upstream ATG^1^** | **Strand** |
| --- | --- | --- | --- | --- |
| S000408 | MYB1AT | WAACCA | -2980 | - |
| S000408 | MYB1AT | WAACCA | -2954 | - |
| S000408 | MYB1AT | WAACCA | -2648 | + |
| S000020 | AMYBOX1 | TAACARA | -2635 | - |
| S000181 | MYBGAHV | TAACAAA | -2635 | - |
| S000447 | WRKY71OS | TGAC | -2578 | - |
| S000447 | WRKY71OS | TGAC | -2552 | + |
| S000180 | MYBST1 | GGATA | -2456 | - |
| S000176 | MYBCORE | CNGTTR | -2341 | - |
| S000409 | MYB2CONSENSUSAT | YAACKG | -2341 | + |
| S000167 | MYBPLANT | MACCWAMC | -2261 | - |
| S000408 | MYB1AT | WAACCA | -2258 | - |
| S000020 | AMYBOX1 | TAACARA | -2255 | - |
| S000181 | MYBGAHV | TAACAAA | -2255 | - |
| S000409 | MYB2CONSENSUSAT | YAACKG | -2036 | - |
| S000176 | MYBCORE | CNGTTR | -2036 | + |
| S000447 | WRKY71OS | TGAC | -1998 | - |
| S000447 | WRKY71OS | TGAC | -1894 | + |
| S000176 | MYBCORE | CNGTTR | -1879 | + |
| S000502 | MYBCOREATCYCB1 | AACGG | -1848 | + |
| S000502 | MYBCOREATCYCB1 | AACGG | -1830 | + |
| S000176 | MYBCORE | CNGTTR | -1808 | + |
| S000176 | MYBCORE | CNGTTR | -1779 | + |
| S000502 | MYBCOREATCYCB1 | AACGG | -1748 | + |
| S000502 | MYBCOREATCYCB1 | AACGG | -1730 | + |
| S000180 | MYBST1 | GGATA | -1503 | - |
| S000447 | WRKY71OS | TGAC | -1444 | + |
| S000175 | MYBATRD22 | CTAACCA | -1284 | + |
| S000408 | MYB1AT | WAACCA | -1283 | + |
| S000447 | WRKY71OS | TGAC | -1266 | - |
| S000447 | WRKY71OS | TGAC | -1245 | + |
| S000408 | MYB1AT | WAACCA | -1177 | + |
| S000447 | WRKY71OS | TGAC | -909 | - |
| S000310 | WBBOXPCWRKY1 | TTTGACY | -897 | + |
| S000447 | WRKY71OS | TGAC | -895 | + |
| S000310 | WBBOXPCWRKY1 | TTTGACY | -887 | + |
| S000447 | WRKY71OS | TGAC | -885 | + |
| S000409 | MYB2CONSENSUSAT | YAACKG | -875 | - |
| S000502 | MYBCOREATCYCB1 | AACGG | -875 | - |
| S000176 | MYBCORE | CNGTTR | -875 | + |
| S000447 | WRKY71OS | TGAC | -804 | - |
| S000021 | AMYBOX2 | TATCCAT | -667 | - |
| S000180 | MYBST1 | GGATA | -665 | + |
| S000408 | MYB1AT | WAACCA | -636 | + |
| S000447 | WRKY71OS | TGAC | -602 | - |
| S000408 | MYB1AT | WAACCA | -579 | + |
| S000447 | WRKY71OS | TGAC | -558 | + |
| S000167 | MYBPLANT | MACCWAMC | -169 | - |
| S000408 | MYB1AT | WAACCA | -166 | - |
| S000176 | MYBCORE | CNGTTR | -140 | - |
| S000409 | MYB2CONSENSUSAT | YAACKG | -140 | + |
| S000502 | MYBCOREATCYCB1 | AACGG | -139 | + |
| S000447 | WRKY71OS | TGAC | -123 | - |
| S000177 | MYB2AT | TAACTG | -68 | - |
| S000409 | MYB2CONSENSUSAT | YAACKG | -68 | - |
| S000176 | MYBCORE | CNGTTR | -68 | + |

A 3,000bp genomic sequence upstream the ATG of Lsat_1_v5_gn_1_123521 was analysed by NEW PLACE (https://integbio.jp/dbcatalog/en/record/nbdc00168). Putative binding sites for MYB and WRKY transcription factors are grey- and white-shaded, respectively. **1**, Distance in base pairs (bp) before the transcription start codon.
