# Supplementary material for: Transcriptome driven characterization of curly- and smooth-leafed endives reveals molecular differences in the sesquiterpenoid pathway
Source: Hortic Res. 2019 Jan 1;6:1. doi: 10.1038/s41438-018-0066-6 (PMC6312536; doi:10.1038/s41438-018-0066-6)
Supplement: Supplementary file 3 — Supplementary Figures 1–6 [file 41438_2018_66_MOESM3_ESM.pptx]

## Slide 1
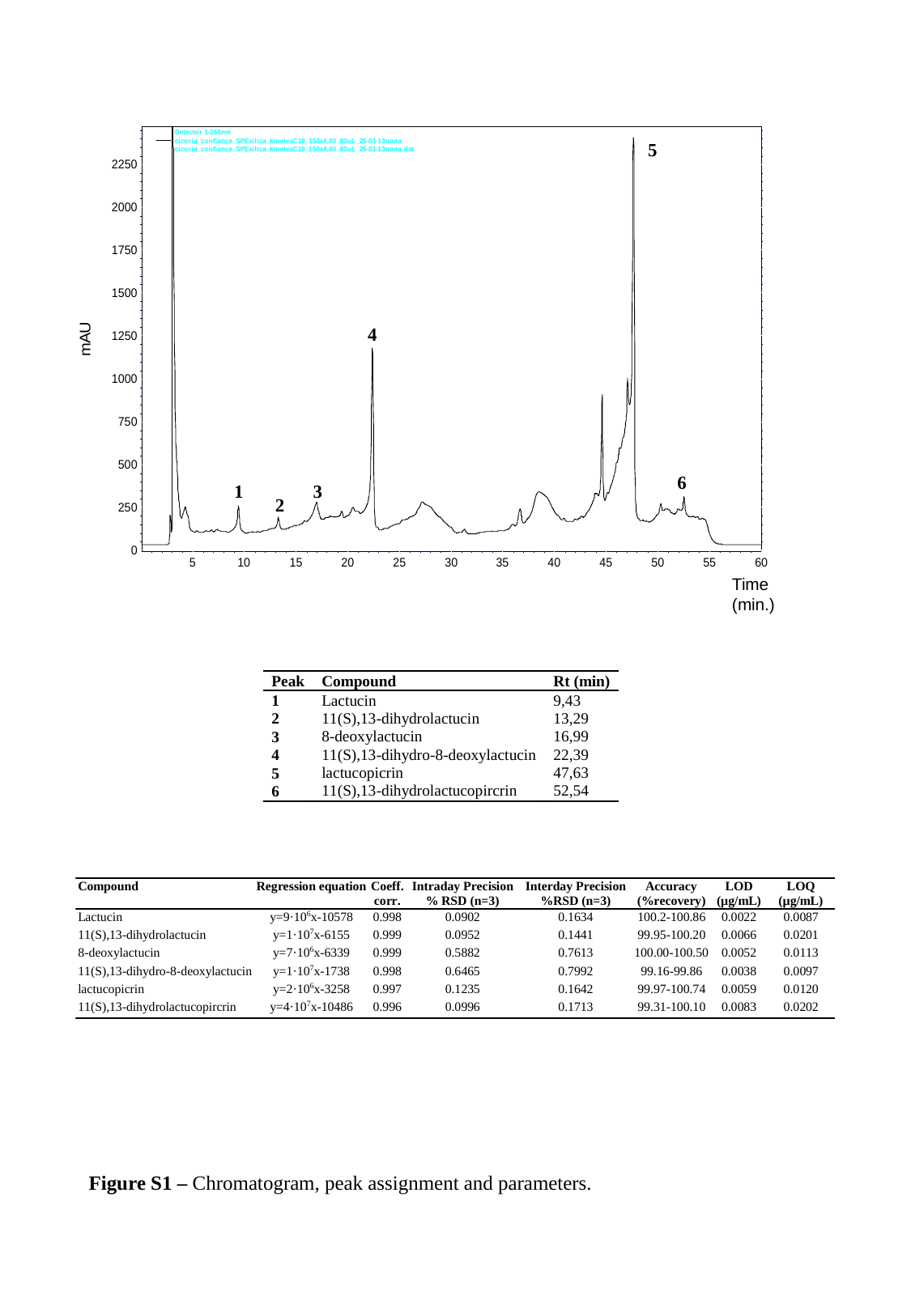

5
4
6
3
1
2
Time (min.)
Figure S1 – Chromatogram, peak assignment and parameters.

## Slide 2
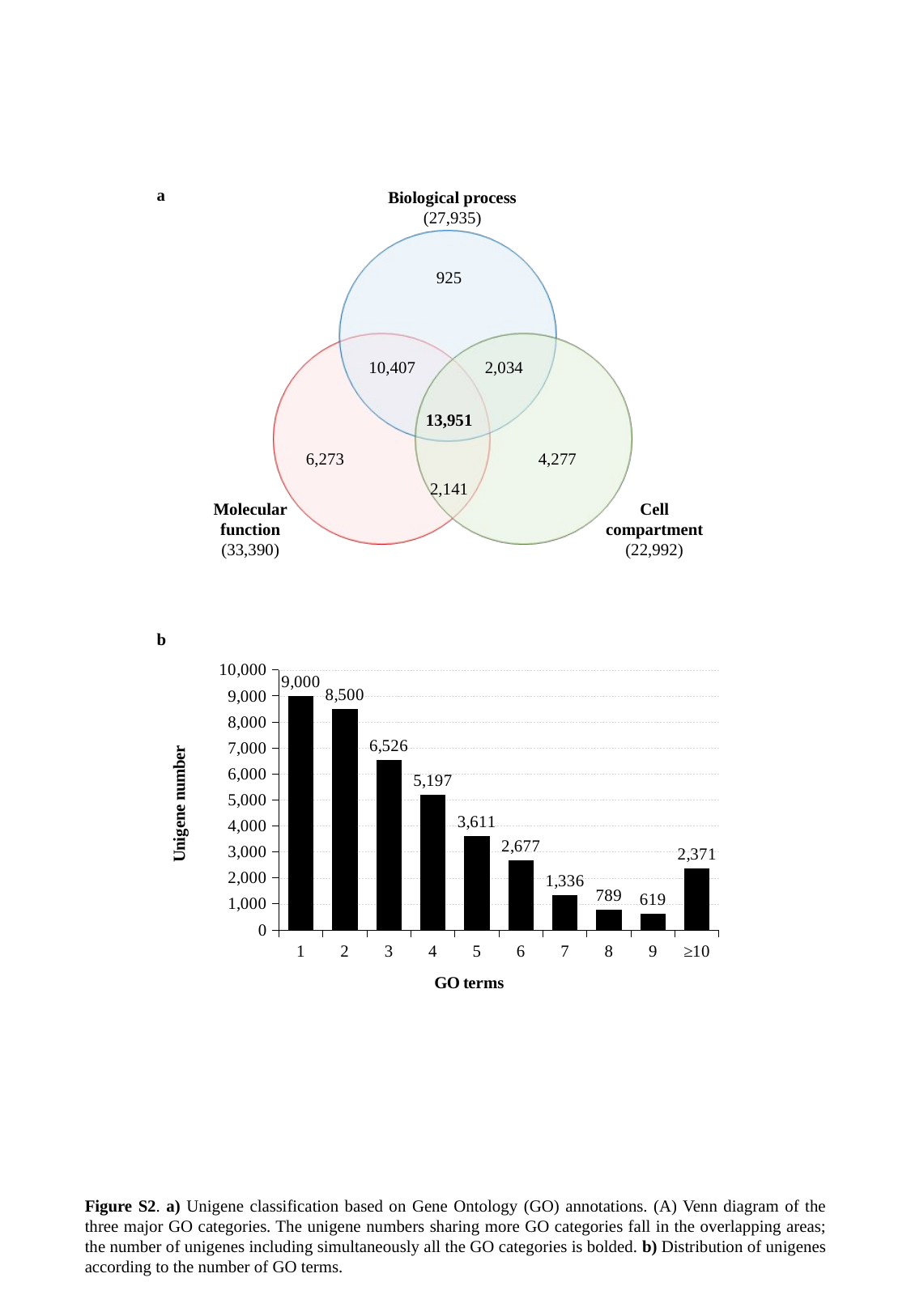

a
Biological process
(27,935)
925
10,407
2,034
13,951
6,273
4,277
2,141
Molecular function
(33,390)
Cell compartment
(22,992)
b
### Chart
| Category | |
|---|---|
| 1 | 9000.0 |
| 2 | 8500.0 |
| 3 | 6526.0 |
| 4 | 5197.0 |
| 5 | 3611.0 |
| 6 | 2677.0 |
| 7 | 1336.0 |
| 8 | 789.0 |
| 9 | 619.0 |
| ≥10 | 2371.0 |Figure S2. a) Unigene classification based on Gene Ontology (GO) annotations. (A) Venn diagram of the three major GO categories. The unigene numbers sharing more GO categories fall in the overlapping areas; the number of unigenes including simultaneously all the GO categories is bolded. b) Distribution of unigenes according to the number of GO terms.

## Slide 3
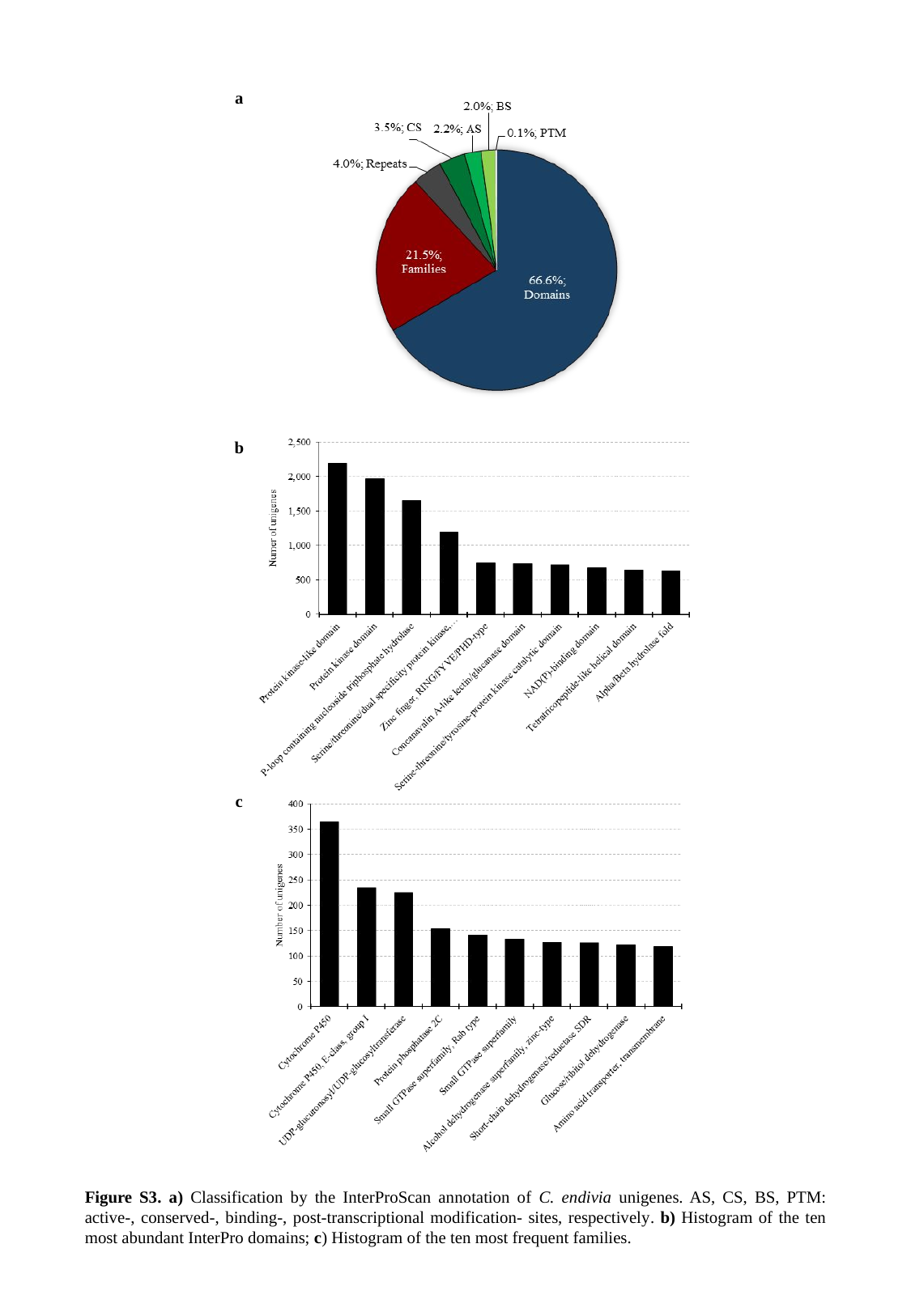

a
b
c
Figure S3. a) Classification by the InterProScan annotation of C. endivia unigenes. AS, CS, BS, PTM: active-, conserved-, binding-, post-transcriptional modification- sites, respectively. b) Histogram of the ten most abundant InterPro domains; c) Histogram of the ten most frequent families.

## Slide 4
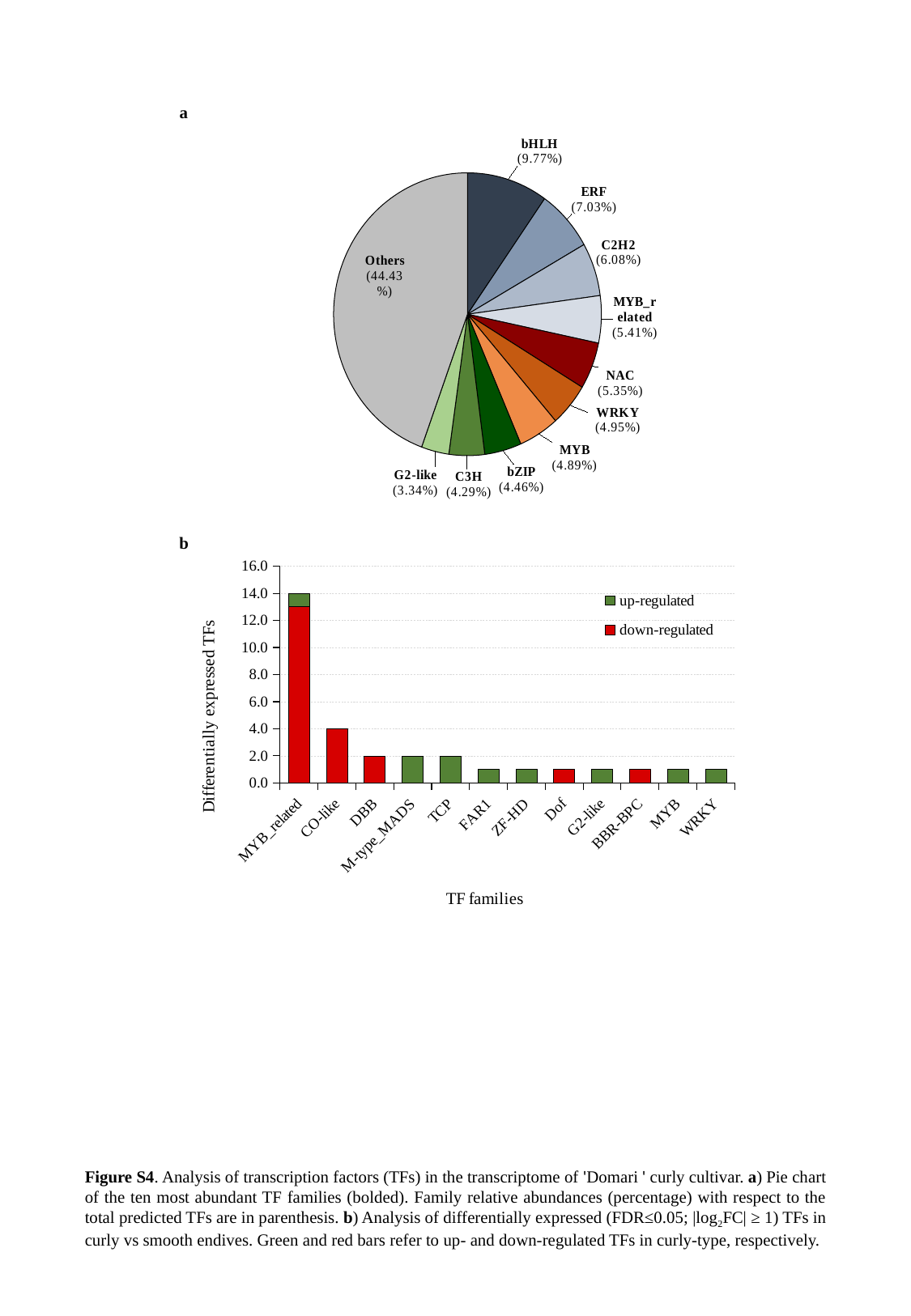

a
### Chart
| Category | | |
|---|---|---|
| bHLH | 535.0 | 0.0976990504017531 |
| ERF | 385.0 | 0.07030679327976626 |
| C2H2 | 333.0 | 0.060810810810810814 |
| MYB_related | 296.0 | 0.05405405405405406 |
| NAC | 293.0 | 0.05350620891161432 |
| WRKY | 271.0 | 0.04948867786705625 |
| MYB | 268.0 | 0.04894083272461651 |
| bZIP | 244.0 | 0.04455807158509861 |
| C3H | 235.0 | 0.042914536157779404 |
| G2-like | 183.0 | 0.03341855368882396 |
| Others | 2433.0 | 0.44430241051862673 |b
### Chart
| Category | | |
|---|---|---|
| MYB_related | 13.0 | 1.0 |
| CO-like | 4.0 | 0.0 |
| DBB | 2.0 | 0.0 |
| M-type_MADS | 0.0 | 2.0 |
| TCP | 0.0 | 2.0 |
| FAR1 | 0.0 | 1.0 |
| ZF-HD | 0.0 | 1.0 |
| Dof | 1.0 | 0.0 |
| G2-like | 0.0 | 1.0 |
| BBR-BPC | 1.0 | 0.0 |
| MYB | 0.0 | 1.0 |
| WRKY | 0.0 | 1.0 |Figure S4. Analysis of transcription factors (TFs) in the transcriptome of 'Domari ' curly cultivar. a) Pie chart of the ten most abundant TF families (bolded). Family relative abundances (percentage) with respect to the total predicted TFs are in parenthesis. b) Analysis of differentially expressed (FDR≤0.05; |log2FC| ≥ 1) TFs in curly vs smooth endives. Green and red bars refer to up- and down-regulated TFs in curly-type, respectively.

## Slide 5
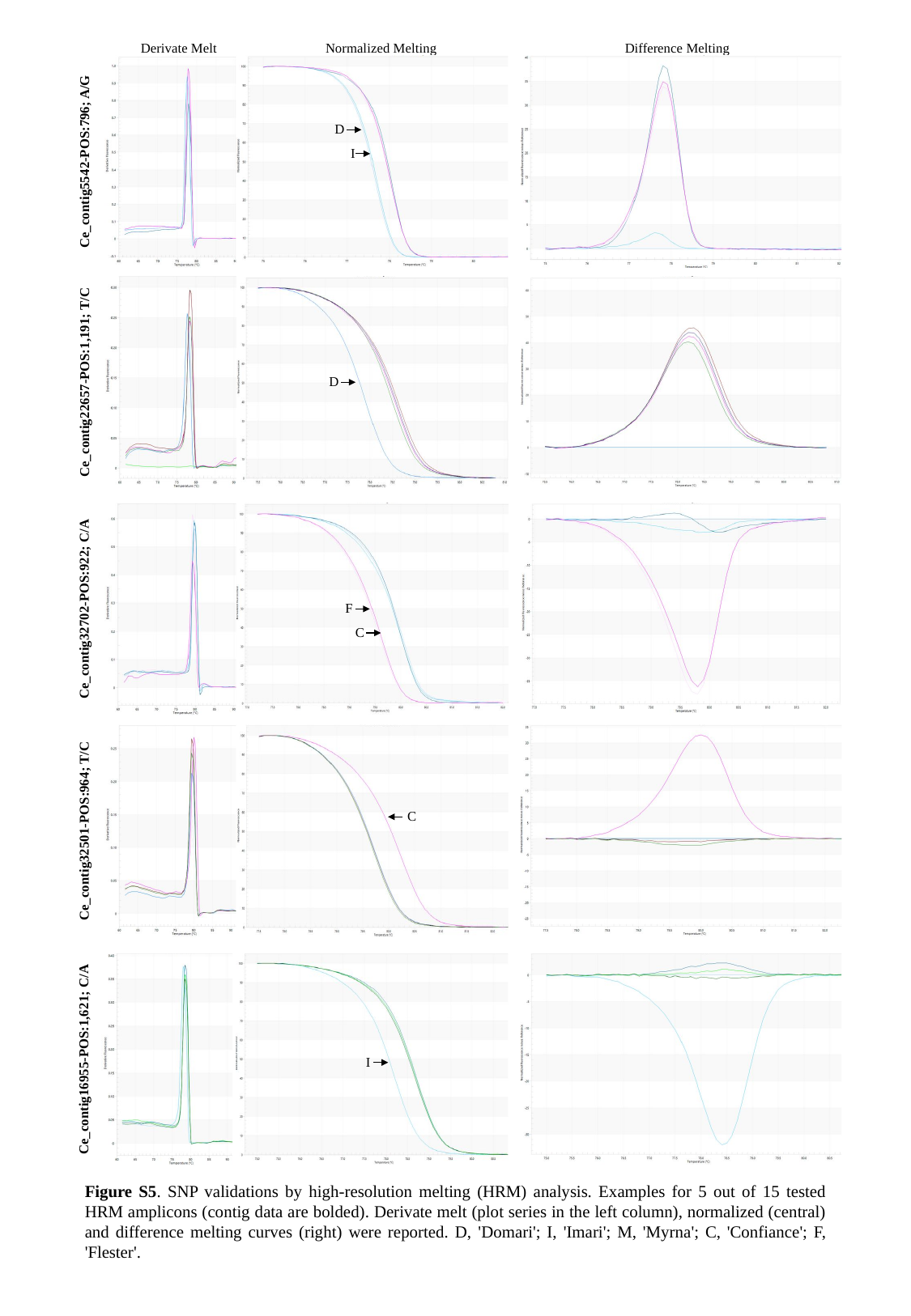

Derivate Melt
Normalized Melting
Difference Melting
Ce_contig5542-POS:796; A/G
D
I
Ce_contig22657-POS:1,191; T/C
D
Ce_contig32702-POS:922; C/A
F
C
C
Ce_contig32501-POS:964; T/C
Ce_contig16955-POS:1,621; C/A
I
Figure S5. SNP validations by high-resolution melting (HRM) analysis. Examples for 5 out of 15 tested HRM amplicons (contig data are bolded). Derivate melt (plot series in the left column), normalized (central) and difference melting curves (right) were reported. D, 'Domari'; I, 'Imari'; M, 'Myrna'; C, 'Confiance'; F, 'Flester'.

## Slide 6
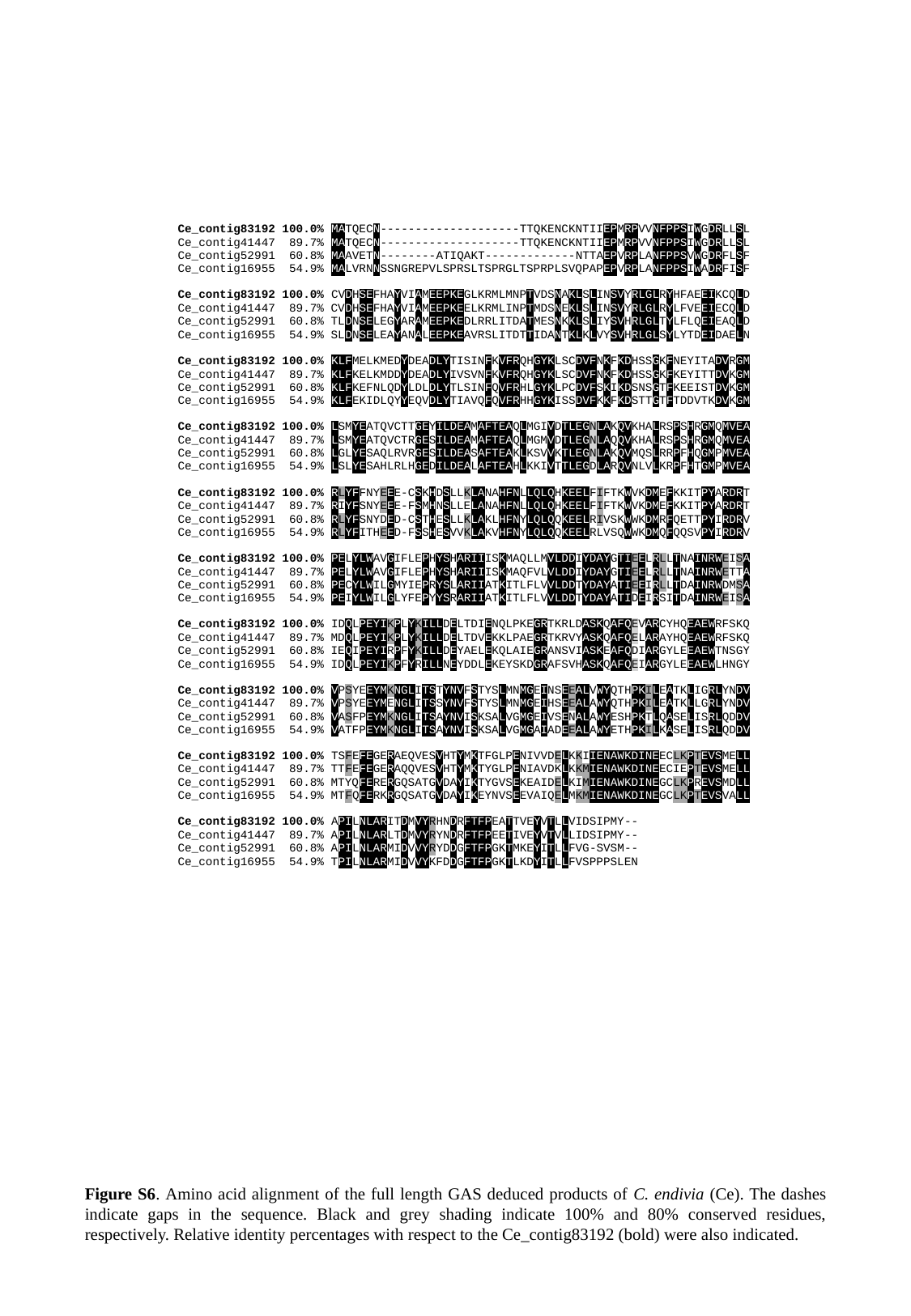

Figure S6. Amino acid alignment of the full length GAS deduced products of C. endivia (Ce). The dashes indicate gaps in the sequence. Black and grey shading indicate 100% and 80% conserved residues, respectively. Relative identity percentages with respect to the Ce_contig83192 (bold) were also indicated.
